# Optimizing extracellular vesicles’ isolation from chronic lymphocytic leukemia patient plasma and cell line supernatant

**DOI:** 10.1172/jci.insight.137937

**Published:** 2021-08-09

**Authors:** Sara Elgamal, Emanuele Cocucci, Ellen J. Sass, Xiaokui M. Mo, Angela R. Blissett, Edward P. Calomeni, Kerry A. Rogers, Jennifer A. Woyach, Seema A. Bhat, Natarajan Muthusamy, Amy J. Johnson, Karilyn T. Larkin, John C. Byrd

**Affiliations:** 1 Division of Hematology, Department of Internal Medicine, College of Medicine,; 2 Comprehensive Cancer Center,; 3 Division of Pharmaceutics, College of Pharmacy,; 4 Department of Biomedical Informatics, College of Medicine,; 5 Department of Pathology, College of Medicine, and; 6 College of Veterinary Medicine, The Ohio State University, Columbus, Ohio, USA.

**Keywords:** Hematology, Oncology, Leukemias

## Abstract

In chronic lymphocytic leukemia (CLL) and very likely all cancer types, extracellular vesicles (EVs) are a common mechanism by which intercellular messages are communicated between normal, diseased, and transformed cells. Studies of EVs in CLL and other cancers have great variability and often lack reproducibility. For CLL patient plasma and cell lines, we sought to characterize current approaches used in isolating EV products and understand whether cell culture–conditioned media or complex biological fluids confound results. Utilizing nanoparticle tracking analysis, protein quantification, and electron microscopy, we show that ultracentrifugation with an OptiPrep cushion can effectively minimize contaminants from starting materials including plasma and conditioned media of CLL cell lines grown in EV-depleted complete RPMI media but not grown in the serum-free media AIM V commonly used in CLL experimental work. Moreover, we confirm the benefit of including 25 mM trehalose in PBS during EV isolation steps to reduce EV aggregation, to preserve function for downstream applications and characterization. Furthermore, we report the highest particles/**μ**g EVs were obtained from our CLL cell lines utilizing the CELLine bioreactor flask. Finally, we optimized a proliferation assay that offers a functional evaluation of our EVs with minimal sample requirements.

## Graphical abstract

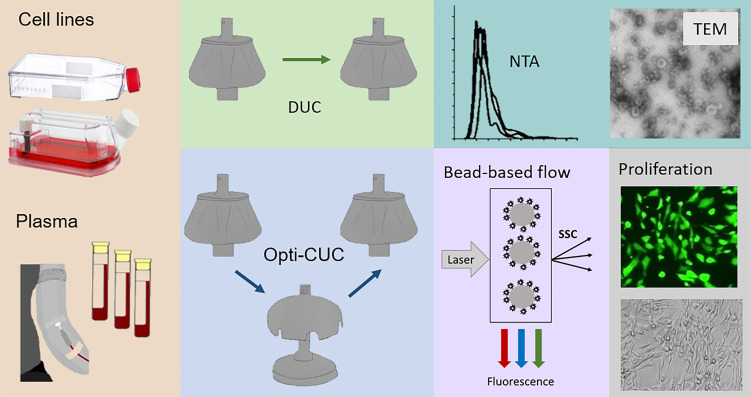

## Introduction

Extracellular vesicles (EVs) are a diverse group of membranous nanoparticles produced by normal, diseased, and neoplastic cells. EVs carry DNA, RNAs, and membrane and soluble proteins (1–4). As such, EVs mediate the transfer of their biologically active cargo molecules, offering an intercellular means of communication (5). Tumor-associated EVs have been implicated in cancer progression via microenvironment modulation and immune suppression (6, 7). We have previously shown that chronic lymphocytic leukemia (CLL) plasma–derived exosomes have a distinct microRNA signature, with miR-150, miR-155, and miR-29 family upregulated but miR-223 downregulated compared with healthy donors (8). With the great promise of EV research, it is critical to robustly isolate sufficient amounts of EVs in a reproducible manner, making standardization of isolation techniques a high-priority task.

Several purification methods are utilized for EV isolation. The gold standard and most widely used technique is differential ultracentrifugation (DUC; ref. 9). DUC generates EVs of relatively homogenous size populations via a series of centrifugation steps with increasing speeds by which intact cells, dead cells, cell debris, and large EVs are eliminated (10, 11). Alternative methods of EV isolation include immunoaffinity-based methods, polymer-based precipitation techniques, or size-exclusion chromatography (SEC; ref. 2). Immunoaffinity and precipitation methods have significant drawbacks for downstream analysis of the EV products. SEC maintains functional and morphological integrity of EVs, since it separates EVs from other biomolecules by size (12–14), and is routinely used to isolate EVs from biological samples with small starting volumes (13, 15–17). The large volume of starting material is a significant drawback when isolating EVs from conditioned cell culture media (CCM; refs. 14, 18).

Density gradient ultracentrifugation is one of the best methods for EV isolation, offering a higher purity in comparison with classic DUC (9, 19, 20). Iodixanol (provided as a 60% solution from MilliporeSigma under the name OptiPrep) is superior to sucrose for density gradient since it can form isosmotic solutions at different densities and thus preserves vesicle size and shape and isolates EVs devoid of virions (21, 22). Another common approach is a 2-step isolation method where ultracentrifugation (UC) is followed by a density gradient cushion. For this purpose 30% sucrose density cushion has been used (23–25). This cushion has a density of 1.12 to 1.18, which is equivalent to that of exosomes (1.15–1.19 g/mL); thus, it can separate protein contaminants of higher density while maintaining exosomes’ integrity by its cushioning effect (23). Similarly, 17% iodixanol is also used (26, 27).

EV isolation from complex biological fluids is very challenging. For plasma, the dynamic range in protein concentration spans at least 10 orders of magnitude (28), with the most abundant protein, albumin, at approximately 50 mg/mL compared with cytokines of low abundance, such as interleukin-6 at approximately 5 pg/mL (29, 30). Although EV isolation from cell line CCM may seem less complex, the culture media of choice can have downstream implications. Most cells are cultured in the presence of fetal bovine serum (FBS) to support optimal growth. This introduces contaminating bovine EVs as well as other macromolecular complexes (31–33). Bovine EVs can be depleted from complete media before usage by performing overnight ultracentrifugation (34) or by purchasing commercially available EV-depleted FBS (14, 35). Furthermore, this EV-depleted media can induce cell stress and alter EV release (18, 36). Another approach used to avoid FBS contaminations is the substitution of FBS by chemically defined media supplements that can maintain optimized cell proliferation and survival (33) or replacing FBS-supplemented media altogether with serum-free media like AIM V (26, 37–39). Due to the ability of AIM V media to maintain CLL cell survival ex vivo, this is commonly used in CLL biologic studies.

In recent work, Auber et al. raised serious concerns about miRNA contamination associated with the chemically defined supplement NS21 (33). The authors assessed for the first time 3 defined culture media supplements (NS16, NS19, and NS21) and reported that subjecting unconditioned media with NS21 supplement to the EV isolation workflow resulted in pellets with EV miRNAs, raising serious concerns about unknown media-derived contaminants (33).

The variation in EV product is attributed not only to different isolation methods but also to different handling of the isolated pellet. Some EV isolation protocols include an incubation step with the reducing agent dithiothreitol (DTT) to minimize nonvesicular protein contamination (40, 41). Interestingly, Santucci et al. recently demonstrated that the use of DTT above 37°C generates massive protein aggregations, which could be the result of disulfide bridges forming between EVs and surrounding macromolecules (42). Another reagent that has been used in the EV isolation process is trehalose. This is a natural sugar commonly used as a (cryo-) preservative for vaccines, labile protein drugs, and liposomes (43, 44). Toxicity studies established its safety in humans for oral, gastric, or parenteral administration (45, 46). Its bioprotective abilities include preventing protein aggregation; stabilizing proteins, cell membranes, and liposomes, and decreasing intracellular ice formation upon freezing (47). Trehalose has been used to reduce loss of exosomes during freeze-drying (patent CN104488850A). Bosch et al. demonstrated the benefits of 25 mM trehalose in PBS (PBS/Tre) to maintain dispersal and functionality of β cell exosome-like vesicles (48).

Beyond the issue of heterogeneity in EV preparation, there are several other hurdles standing in the way of successful translation of EVs to clinical application (49, 50), including the lack of quality control criteria and the need to follow regulatory guidelines to allow clinical testing (51). Therefore it is necessary to define methods to assess EV functionality (50, 52, 53). Functional assays are capable of assessing molecular and physiological effects of an EV preparation on target cells. While this effect does not have to be linked to the potential of this EV to cause a specific therapeutic effect, it assesses the potential of an EV preparation to elicit a quantifiable effect. Functional assays offer a means to help the EV global community to compare research and assess the quality, efficacy, and therapeutic dose of EVs. In this work, we report an optimized proliferation assay (26, 54) where we have utilized a stromal cell line with GFP fluorescence (HS5-GFP) that can allow us to monitor the effect of EVs at several time points with minimal sample requirements.

## Results

### Collection and processing.

**Figure 1:**
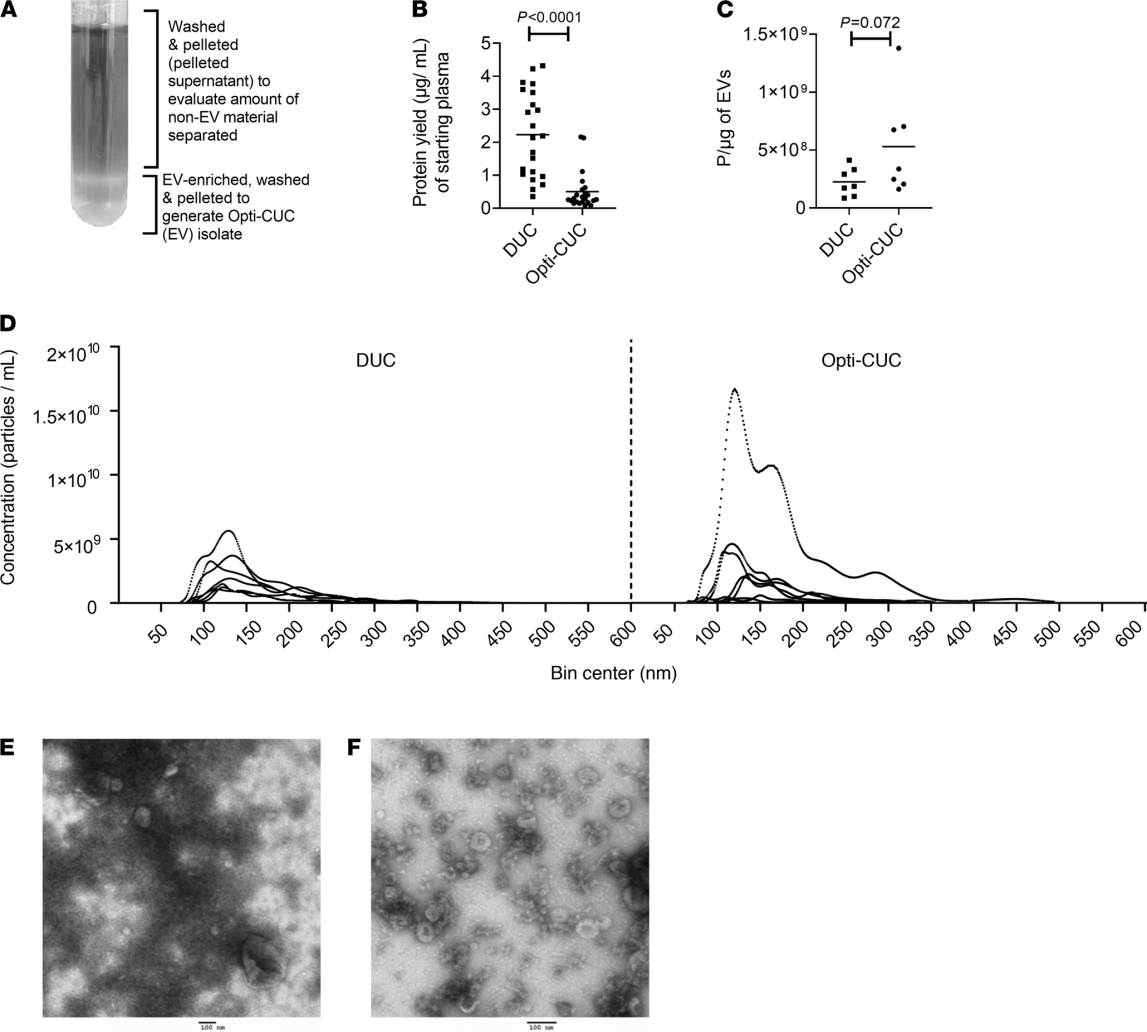
CLL plasma–derived EVs isolated by DUC and Opti-CUC. (**A**) Diagram of Opti-CUC tube after 100,000*g* spin. (**B**) Protein yield (μg) per milliliter starting plasma volume for samples (*n* = 22, 2-sample 2-tailed *t* test, *P* < 0.0001) isolated by DUC or Opti-CUC. Horizontal line represents mean. (**C**) Particle/μg value for EV samples in panel **D** (*n* = 7, 2-sample 2-tailed *t* test, *P* = 0.072). Horizontal line represents mean. (**D**) Isolated particles subjected to nanoparticle tracking analysis (NTA). *n* = 7. Measurement for concentration and size distribution. A representative plot presented is the average of three 30-second videos. (**E** and **F**) Representative electron microscopy images of DUC isolated EVs (**E**) and Opti-CUC isolated EVs (**F**). Scale bar: 100 nm. Electron microscopy performed at least 3 times for each isolation method.

EVs were isolated from the plasma of patients with CLL using 2 methods. The first was the classic DUC (2, 9, 12, 34). Blood samples were spun at 300*g* for 10 minutes at room temperature to separate plasma. To remove platelets, plasma was subjected to a 2500*g* spin for 15 minutes twice at room temperature (55, 56) before storage at –80°C. Upon thawing, plasma was diluted 1:1 with cold PBS. After this dilution, all following centrifugation steps were conducted at 4°C. Following plasma dilution, a 2000*g* spin step for 20 minutes was performed to remove any debris generated during thawing. Larger vesicles were removed by a 10,000*g* step for 30 minutes (57, 58). This supernatant was spun at 100,000*g* for 70 minutes to get the EV-enriched pellet. Finally, this pellet was suspended in 25 mM PBS/Tre (48), and the 100,000*g* spin was repeated to wash non-EV proteins. The second method we refer to as OptiPrep Cushioned-UC (Opti-CUC). This method follows the same steps of DUC with an additional UC step at 100,000*g* for 75 minutes over a 17% OptiPrep cushion (26, 27). This additional step is applied before the final 100,000*g* wash spin. Because 17% OptiPrep cushion has a density close to exosomes and other small EVs, during UC the exosomes can float away from other vesicles and contaminants and remain in the cushion while contaminants are pelleted (27). The interphase and lower phase are collected and washed in a final 100,000*g* spin for 70 minutes to generate Opti-CUC EV isolate (Figure 1A). To compare these methods, we isolated EVs from 22 CLL patients by DUC and another 22 by Opti-CUC. Evaluation of protein yield showed an average of 2.23 ± 1.29 and 0.50 ± 0.57 μg/mL of starting plasma for DUC and Opti-CUC, respectively (Figure 1B). Nanoparticle tracking analysis (NTA) revealed (Figure 1, C and D) an average concentration of EVs of 2.19 × 10^11^ ± 9.49 × 10^9^ and 3.43 × 10^11^ ± 1.20 × 10^10^ particles/mL for DUC and Opti-CUC, respectively. Utilizing this simple calculation of particle count to protein yield as previously described (20) as a proxy of sample purity, EV isolates of these methods showed an average 2.26 × 10^8^ ± 1.22 × 10^8^ and 5.3 × 10^8^ ± 4.34 × 10^8^ particles/μg for DUC and Opti-CUC, respectively. Electron microscopy images of DUC and Opti-CUC (Figure 1, E and F) contained cup-shaped vesicles of morphology and size of EVs, where the DUC EVs appeared to have more nonvesicular components. To further evaluate the contribution of the OptiPrep cushion in reducing coisolated contaminants, we evaluated the protein content of the supernatant above the EV-enriched cushion and detected that the pelleted supernatant had an average of 0.98 ± 0.63 μg/mL of starting plasma volume (Supplemental Figure 1A; supplemental material available online with this article; https://doi.org/10.1172/jci.insight.137937DS1). Comparing the Opti-CUC EVs to the corresponding pelleted supernatant by electron microscopy (Supplemental Figure 1B) supported the ability of Opti-CUC to separate nonvesicular material. In accordance with others (26, 27), our results confirm the ability of Opti-CUC to reduce the coisolation of nonvesicular contamination.

### Impact of composition of buffers used during EV isolation.

In this work we used 25 mM PBS/Tre for all wash steps and for dissolving the final pellet (48). We further wanted to investigate if utilizing trehalose earlier in the isolation protocol could improve the quality and yield of the EV isolates, given its ability to reduce aggregation. This is not only a problem while dissolving the final EV pellet; aggregation has been reported in earlier steps of EV isolation (19, 34, 59–61), and the 10,000*g* centrifugation step can remove aggregates of the desired smaller vesicles. Given the total volume limitation from an individual patient, it was not feasible to do comparative studies by splitting the small volumes of plasma we could obtain per sample (10–20 mL), and so to overcome this, we proceeded with pooled plasma to be able to evaluate workflows simultaneously and minimize variability. Trehalose was added at the stage of diluting plasma 1:1 with PBS directly after thawing. Hence, plasma pools were split among 3 workflows: a) Opti-CUC where plasma was diluted with equal volume of PBS, b) Opti-CUC-Tre where plasma was diluted with equal volume of 50 mM trehalose in PBS, and c) DUC where plasma was diluted with equal volume PBS and processed by standard DUC (absent of any cushion).

**Figure 2:**
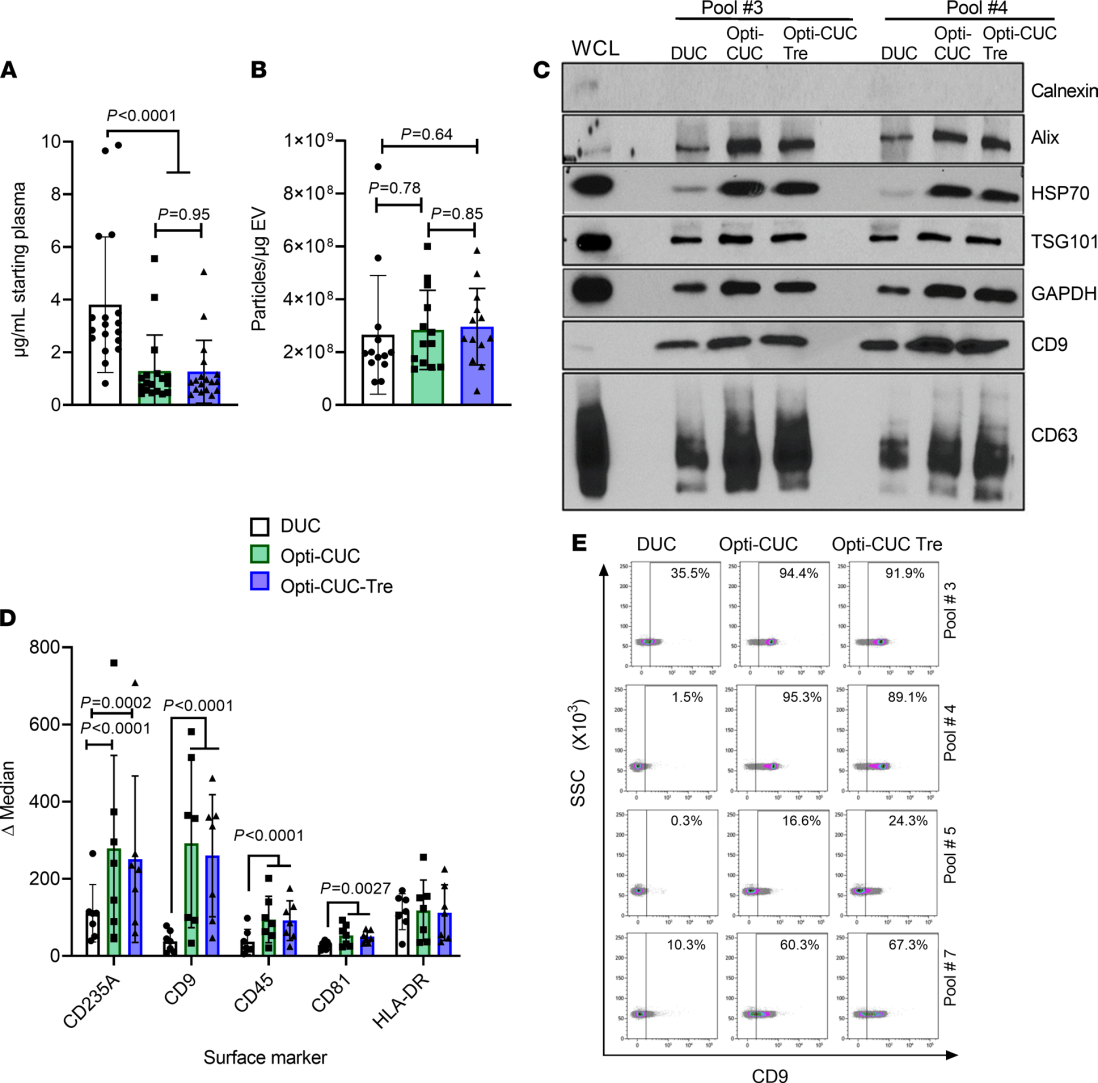
EVs isolated from CLL plasma pools subjected to EV isolation by the 3 methods, DUC, Opti-CUC and Opti-CUC-Tre. (**A**) Protein yield (μg) per milliliter starting plasma volume. *n* = 18. (**B**) P/μg value for some of the EV samples in panel **A** (*n* = 13). Some of these NTA analysis plots are in Supplemental Figure 3. (**C**) A representative Western blot analysis of 2 sets of plasma pool–EV isolates prepared by the 3 methods. Western blots were done for 4 sets. WCL, whole cell lysate. (**D**) Bead-based flow cytometry analysis of EV isolates from the plasma pools. Delta median fluorescence of samples calculated by subtracting median fluorescence intensity (MFI) of each sample from its isotype control (*n* = 7). For graphs **A**, **B**, and **D**, 2-way ANOVA and data are represented as mean ± SD. (**E**) Representative density plots of CD9 fluorescence for 4 of the plasma pools in **D**. Each row shows the DUC, Opti-CUC, or Opti-CUC-Tre plot for a plasma pool. Gates set by isotypes.

All plasma pools showed higher protein yield for the DUC condition in comparison with both Opti-CUC conditions (Figure 2A and Supplemental Figure 2A), with an average of 3.80 ± 2.57, 1.28 ± 1.37, and 1.26 ± 1.19 μg/mL of starting plasma for DUC, Opti-CUC, and Opti-CUC-Tre, respectively (Figure 2A). The calculated particles/μg (P/μg) average was 2.65 × 10^8^ ± 2.25 × 10^8^, 2.84 × 10^8^ ± 1.50 × 10^8^, and 2.96 × 10^8^ ± 1.45 × 10^8^ for DUC, Opti-CUC, and Opti-CUC-Tre, respectively (Figure 2B). Looking at each plasma pool individually (Supplemental Figure 2B), most sets showed a lower P/μg ratio for the DUC condition. The use of only particles/protein ratio as a purity metric may not suffice in this comparison since there is a difference in the specificity of these isolation methods. It is recommended for EVs recovered from less specific methods to utilize more than one quantification method to evaluate purity (62) like protein/lipid ratio (63, 64) and RNA/particle ratio (65). According to the recovery versus specificity grid reported in minimal information for studies of EVs (MISEV) 2018 update (62), DUC is a method with intermediate recovery and intermediate specificity where recovered EVs can be contaminated with lipoproteins, ribonucleoproteins, and extravesicular protein complexes/aggregate (2, 12, 16, 36, 62, 66–68). On the other hand, Opti-CUC includes a flotation step on a density gradient medium that reduces contamination by nonvesicular components, making it a method of low recovery and high specificity (62). NTA plots showed a slightly wider range of size distribution for the DUC condition in comparison with the Opti-CUC conditions (Supplemental Figure 3). Figure 2C (see complete unedited blots in the supplemental material) shows a representative Western blot analysis of the 3 methods of isolation (DUC, Opti-CUC, or Opti-CUC-Tre) performed with pooled plasma samples. Both Opti-CUC samples showed enhanced detection of EV markers CD9, CD63, Alix, HSP70, and TSG 101 in comparison with DUC. This shows the benefit of Opti-CUC to reduce nonvesicular contaminant proteins in comparison with DUC. Bead-based flow cytometry (69) showed enhanced surface marker detection of CD235A, CD9, CD45, and CD81 for Opti-CUC and Opti-CUC-Tre versus DUC (Figure 2, D and E, and Supplemental Figure 4).

### MEC1 and OSU-CLL cell line–derived EVs.

**Figure 3:**
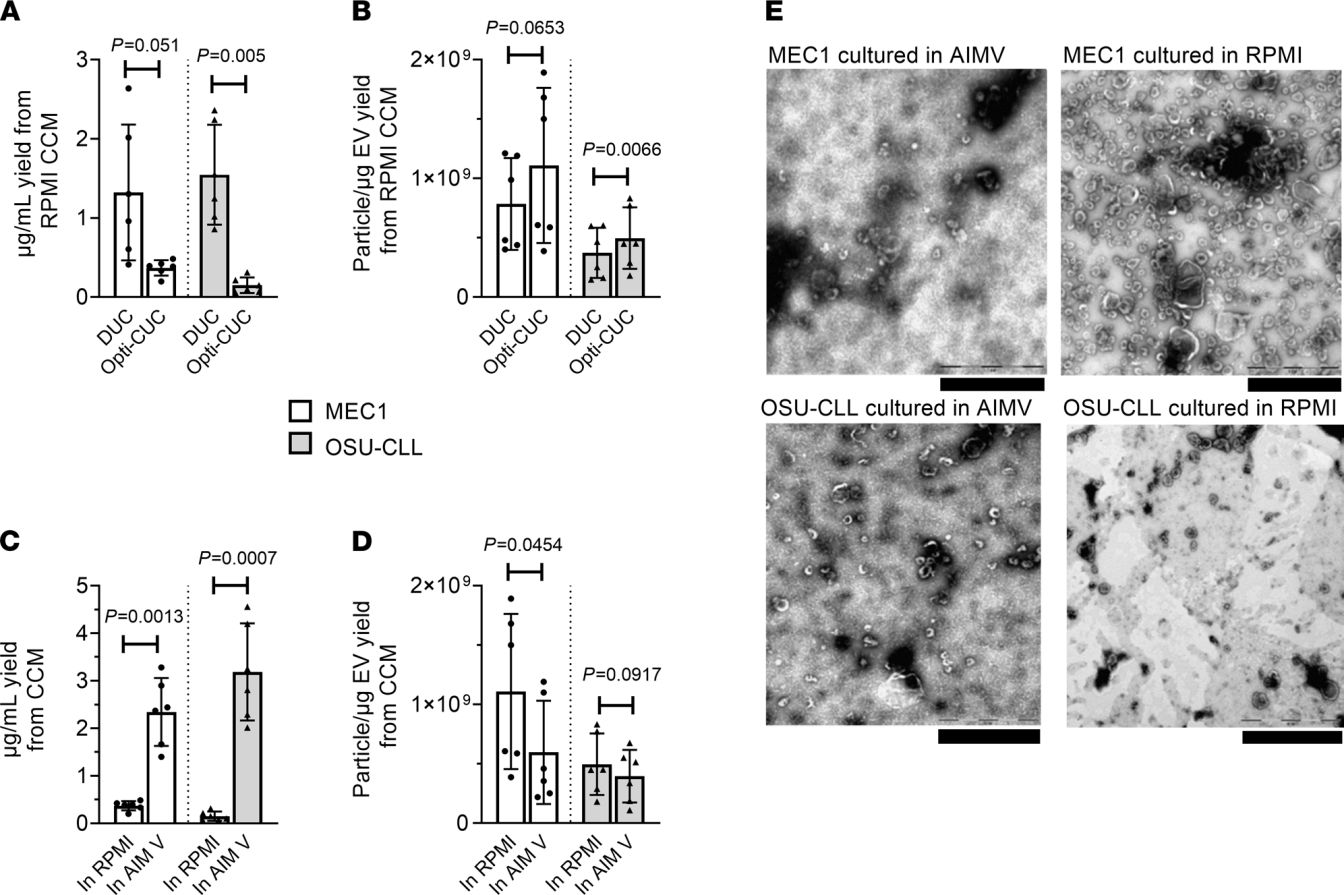
CCM for the CLL cell lines MEC1 and OSU-CLL. (**A**) Protein yield (μg) per milliliter starting RPMI CCM by Opti-CUC or DUC. *P* = 0.051 and 0.005 for MEC1 and OSU-CLL, respectively. (**B**) P/μg value for EV isolates in panel **A**. *P* = 0.0653 and 0.0066 for MEC1 and OSU- CLL, respectively. (**C**) Protein yield (μg) per milliliter starting RPMI or AIM V CCM for MEC1 and OSU-CLL processed by Opti-CUC. *P* = 0.0013 and 0.0007 for MEC1 and OSU-CLL, respectively. RPMI data repeated from panel **A** for comparison. (**D**) P/μg value for EV isolates in panel **C**. RPMI data repeated from panel **B** for comparison. For graphs **A**–**D**, data are represented as mean ± SD. *n* = 6. Paired 2-tailed *t* test. (**E**) Electron microscopy images of Opti-CUC purified EVs from MEC1 and OSU-CLL. All scale bars: 1 μm. RPMI, EV-depleted complete RPMI.

**Figure 4:**
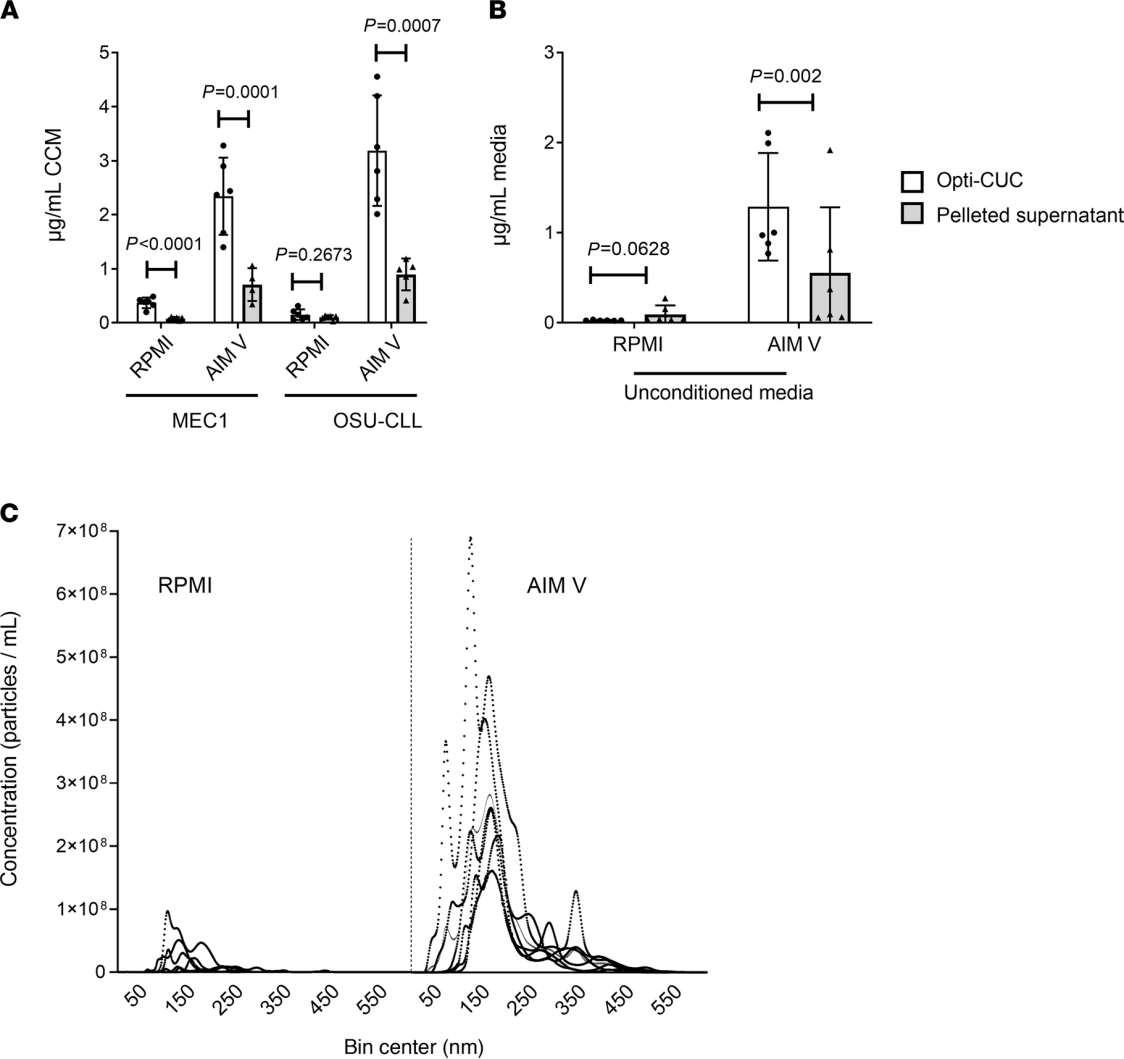
Comparison of RPMI and AIM V culture media. (**A**) Protein yield per milliliter starting media (μg/mL) for MEC1 and OSU-CLL detected in Opti-CUC EV isolate and pelleted supernatant, Opti-CUC EV isolate data repeated from Figure 3C for comparison (*n* = 6, 2-way ANOVA). (**B**) Protein yield per milliliter starting media (μg/mL) of AIM V and RPMI unconditioned media (*n* = 6, 2-way ANOVA) detected in Opti-CUC isolate and pelleted supernatant. For **A** and **B** data are represented as mean ± SD. (**C**) Isolated particles by Opti-CUC for the unconditioned media shown in panel **B** — subjected to NTA measurement for concentration and size distribution. A representative plot presented is the average of three 30-second videos, *n* = 6.

MEC1 (70) and OSU-CLL (71) are 2 well characterized cell lines derived from patients with CLL. We compared DUC and Opti-CUC for the 2 CLL cell lines (MEC1 and OSU-CLL) utilizing EV-depleted complete RPMI. The average protein yield (μg/mL of CCM) for Opti-CUC was significantly less than that for DUC: 0.36 ± 0.09 versus 1.32 ± 0.85 and 0.14 ± 0.10 versus 1.54 ± 0.63 μg/mL for MEC1 and OSU-CLL, respectively (Figure 3A). On the other hand, the P/μg for the Opti-CUC was higher than DUC: 1.11 × 10^9^ ± 6.52 × 10^8^ versus 7.84 × 10^8^ ± 3.86 × 10^8^ and 4.96 × 10^8^ ± 2.59 × 10^8^ versus 3.72 × 10^8^ ± 2.11 × 10^8^ P/μg for MEC1 and OSU-CLL, respectively (Figure 3B and Supplemental Figure 5). This confirms that higher protein yields are not directly correlated to higher EV yields and demonstrated the ability of Opti-CUC to separate out non-EV particles for cell line CCM. We then evaluated the serum-free media AIM V, a proprietary serum-free medium that was developed in 1987 to support adoptive immunotherapy (72) and proposed as optimal for incubation of CLL cells in vitro. Because it is devoid of bovine proteins, this medium has research and clinical applications (25). It has also been used for EV production (26, 37–39). It is prepared from a master formulation of DMEM, HEPES buffer, human serum albumin, human transferrin, and cholesterol. Comparing protein yield (μg/mL starting CCM) of EV isolates produced from EV-depleted complete RPMI in comparison with AIM V cultures showed a significant increase: 0.36 ± 0.1 versus 2.34 ± 0.71 and 0.14 ± 0.10 versus 3.18 +1.02 for MEC1 and OSU-CLL, respectively (Figure 3C). The P/μg ratio for EV-depleted RPMI was higher than AIM V: 1.11 × 10^9^ ± 6.52 × 10^8^ versus 5.97 × 10^8^ ± 4.36 × 10^8^ and 4.96 × 10^8^ ± 2.59 × 10^8^ versus 3.96 × 10^8^ ± 2.21 × 10^8^ P/μg for MEC1 and OSU-CLL, respectively (Figure 3D and Supplemental Figure 5A). Electron microscopy images of EVs isolated from both AIM V and EV-depleted complete RPMI by Opti-CUC showed cup-shaped vesicles of morphology and size of EVs; qualitative comparison of the images suggested that RPMI-isolated EVs had fewer nonvesicular components (Figure 3E and Supplemental Figure 6). To evaluate the contribution of the OptiPrep cushion in reducing coisolated contaminants of CCM, we further pelleted the supernatant above the EV-enriched cushion and detected for AIM V pelleted supernatant: 0.70 ± 0.30 and 0.89 ± 0.29 μg/mL CCM for MEC1 and OSU-CLL, respectively; for EV-depleted complete RPMI pelleted supernatant: 0.07 ± 0.03 and 0.10 ± 0.04 μg/mL CCM for MEC1 and OSU-CLL, respectively (Figure 4A). This indicates that EV-depleted complete RPMI had minimal coisolated protein. Upon processing both unconditioned media by Opti-CUC, we found that unconditioned AIM V generated an obvious Opti-CUC isolate while EV-depleted complete RPMI did not. Our protein analysis of this isolate for the unconditioned media showed 1.28 ± 0.59 and 0.02 ± 0.005 μg/mL for AIM V and EV-depleted complete RPMI, respectively (Figure 4B). To evaluate the contribution of the OptiPrep flotation step on reducing the coisolated media protein components, we determined the protein content of the pelleted supernatant and detected 0.55 ± 0.73 and 0.09 ± 0.1 μg/mL for unconditioned AIM V and EV-depleted complete RPMI, respectively; thus the cushion (Opti-CUC isolate) retained approximately 70% and 22% of proteins originating from the unconditioned AIM V and EV-depleted complete RPMI, respectively (Figure 4B). Furthermore, NTA revealed a size distribution similar to EVs for both unconditioned media (Figure 4C), with an average concentration of 3.2 × 10^10^ ± 1.45 × 10^9^ and 2.26 × 10^9^ ± 2.22 × 10^8^ P/mL for unconditioned AIM V and EV-depleted complete RPMI, respectively. Lamparski et al. previously demonstrated that the initially low level of particulate haptoglobin in AIM V can reach mg/mL concentrations in the final product after copurification with exosomes (25). Aggregated haptoglobin can induce undesirable immune responses to serum components (74–77); furthermore, these protein aggregates are 10,000 times more immunogenic than the soluble form (77). Lamparski et al. also reported that only by ultrafiltration through a 500 kDa NMWCO hollow fiber cartridge could they remove the aggregated proteins while a 30% sucrose/deuterium oxide (D_2_O) (98%) cushion failed to separate those aggregates (25). With this work we confirm that a 17% OptiPrep cushion fails to remove AIM V coisolated components and argue against its use as an EV production media for UC-based isolation methods. In this work we have only investigated the coisolated AIM V protein components. It is yet to be explored if this medium also contributes to EV-RNA contamination.

### EV production utilizing CELLine bioreactor flask.

**Figure 5:**
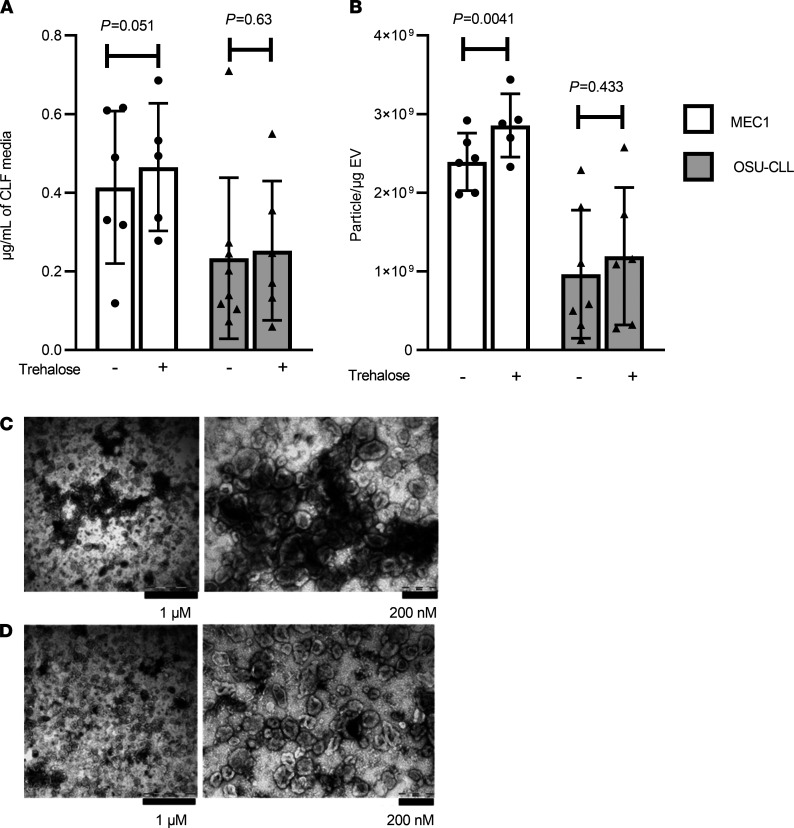
CLF EVs isolated by Opti-CUC in absence (-) or presence (+) of initial trehalose for MEC1 and OSU-CLL cell lines. (**A**) Protein yield (μg) per milliliter total CLF media volume (500 mL). *P* = 0.051 and 0.63 for MEC1 and OSU-CLL, respectively (*n* = 6, paired 2-tailed *t* test). (**B**) P/μg value for CLF EVs in panel **A**. *P* = 0.0041 and 0.433 for MEC1 and OSU-CLL, respectively (*n* = 6, paired 2-tailed *t* test). For graphs **A** and **B**, data are represented as mean ± SD. (**C** and **D**) Representative electron microscopy images of EVs isolated from the MEC1 cell line cultured in the CLF in absence (**C**) or presence (**D**) of initial trehalose. Electron microscopy performed at least 3 times for each isolation method. CLF, CELLine Flask.

**Figure 6:**
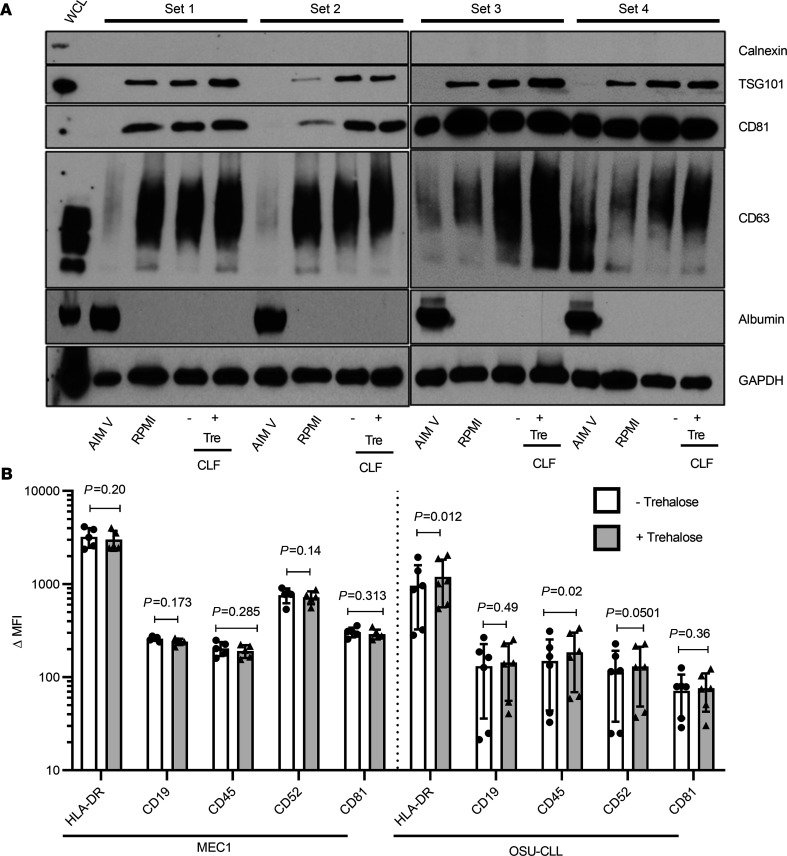
EV isolation from MEC1 and OSU-CLL in standard flasks (cultured in AIM V or RPMI) or CLF (cultured in RPMI). (**A**) Western blot analysis of 4 sets of MEC1 EVs cultured in different media (AIM V or RPMI) in standard flasks and CLF isolated by Opti-CUC in absence (-) or presence (+) of initial trehalose. (**B**) Bead-based flow cytometry analysis of EV isolates from MEC1 and OSU-CLL CLFs in absence (-) or presence (+) of initial trehalose. Delta median fluorescence of samples calculated by subtracting MFI of each sample from its isotype control (*n* = 6, 2-way ANOVA). Data are represented as mean ± SD.

**Figure 7:**
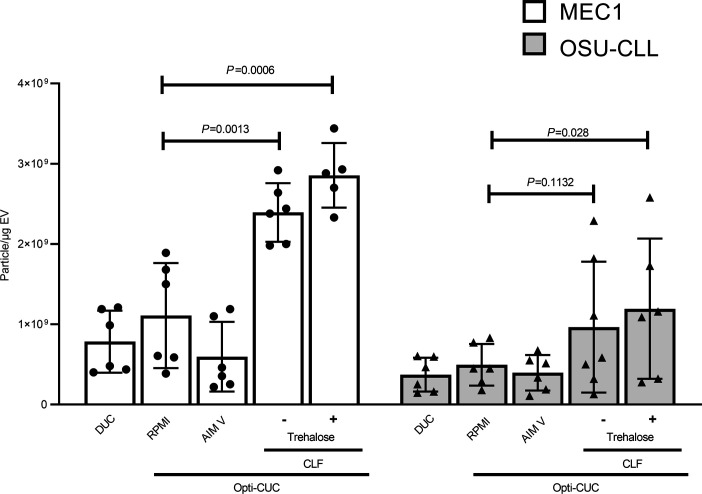
A summary plot showing all P/μg values for all samples reported in this work (n = 6, 2-way ANOVA). Data are represented as mean ± SD.

Classic cell culture flasks permit limited cell numbers and short durations of growth. Scaling up EV production by increasing the number of flasks is time-consuming, costly, and laborious both before the experiment (to prepare the EV-depleted complete RPMI) and after harvesting due to the large amounts of generated CCM. The CELLine bioreactor flask (CLF, Argos Technologies) can address this problem (14, 78). This 2-compartment technology (separated by a 10 kDa semipermeable membrane) allows continuous diffusion of nutrients with simultaneous removal of waste products (27) and thus circumvents the conventional flask restrictions of nutrient depletion and limited oxygen supply (79). The reported dramatic increase in cell number achieved by 3D cell growth also mimics physiological growth conditions, thereby increasing the amount of EVs recovered (78, 80). Wierz et al. demonstrated efficient production of large quantities of MEC1 exosomes utilizing this technology (27). We prepared EVs from our CLL cell lines by Opti-CUC or Opti-CUC-Tre. The average protein yield (μg/mL of CCM) for Opti-CUC-Tre was slightly higher than that for Opti-CUC: 0.46 ± 0.16 versus 0.40 ± 0.20 and 0.25 ± 0.17 versus 0.23 ± 0.24 μg/mL for MEC1 and OSU-CLL, respectively (Figure 5A). The same trend was observed for P/μg where the Opti-CUC-Tre was slightly higher than that for Opti-CUC: 2.86 × 10^9^ ± 4.01 × 10^8^ versus 2.40 × 10^9^ ± 3.64 × 10^8^ and 1.19 × 10^9^ ± 8.72 × 10^8^ versus 1.03 × 10^9^ ± 8.75 × 10^8^ P/μg for MEC1 and OSU-CLL, respectively (Figure 5B). This P/μg increase was significant for MEC1 (*P* = 0.0041) but not for OSU-CLL (*P* = 0.433). Electron microscopy images of Opti-CUC and Opti-CUC-Tre EVs (Figure 5, C and D) contained cup-shaped vesicles of morphology and size of EVs. Qualitative comparison of the images suggests the Opti-CUC-Tre EVs were less packed together than Opti-CUC EVs. Figure 6A (see complete unedited blots in the supplemental material) shows a representative Western blot analysis of 4 sets of MEC1 EV isolates of the different methods and culture vessels discussed. EVs produced from EV-depleted complete RPMI cultures had enriched exosome markers while AIM V–produced EVs showed the least expression of these markers along with notable albumin contamination. CLF samples showed slightly more CD81 and CD63 detection in comparison with regular flask cultures. Bead-based flow cytometry for surface markers known to be present on CLL-derived EVs, such as HLA-DR, CD81, CD19, CD45, and CD52 (9, 81, 82), showed similar levels of expression for MEC1 EVs isolated by Opti-CUC or Opti-CUC-Tre, while OSU-CLL EV isolates showed a borderline increased expression of HLA-DR and CD45 (*P* *=* 0.012 and 0.02, respectively) for Opti-CUC versus Opti-CUC -Tre (Figure 6B). We further investigated if there was a difference in size distribution of the EVs generated by standard flask versus CLF. Indeed we found that the CLF-generated EVs were smaller (*P* < 0.05 for EVs of size 100–400 nm) for both CLL cell lines (Supplemental Figure 7). This is in accordance with very recent work reported by Palviainen et al. (73) noting similar size discrepancy for the prostate cancer cell line PC-3. To this end, we believe EV-depleted complete RPMI is the medium of choice for culturing CLL cell lines. The use of a bioreactor culture vessel offers an EV product of higher yield with Opti-CUC-Tre offering the highest P/μg (Figure 7) and highest P/cell (Supplemental Figure 8) for both CLL cell lines.

### Functional assay for evaluation of EV isolates.

As extensively reviewed by Nguyen et al. (50), several assays with an aspect of proliferation assessment have been developed, many of which are very specific to the cells/biological systems being investigated and dependent on the use of primary cells (83–87). Primary cells have the disadvantages of limited life span, the need for special supplements for their maintenance, and their limited expansion capacity (50). On the other hand, many biological in vitro experiments have also utilized standardized cell lines like the stromal cell line HS5 where EV doses are incubated with these cells and proliferation is assayed by addition of MTS tetrazolium compound after 72 or 96 hours (26, 54). MTS assays can assess cell proliferation, cell viability, and cytotoxicity (88). In this work, we have further optimized this stromal cell–based proliferation assay to use a green fluorescent HS5 (HS5-GFP). This modification offers 3 advantages. First, we read out the assay at several time points, which cannot be achieved by MTS assays without preparing several plates at setup such that 1 plate is dedicated to each time point. Second, GFP absorbance can serve as a proxy for cell number, which would offer additional information to the MTS readout. MTS typically evaluates cell proliferation by assuming stable mitochondrial activity (88), and we can imagine that EV-induced metabolic changes in recipient cells (89–91) could alter that assumption and affect the assay’s reproducibility. Third, GFP absorbance of this fluorescent cell line offers the ability to compare across experiments; in our hands the absorbance of plated HS5-GFP cells for the buffer control wells at time points was comparable across experiments (data not shown).

**Figure 8:**
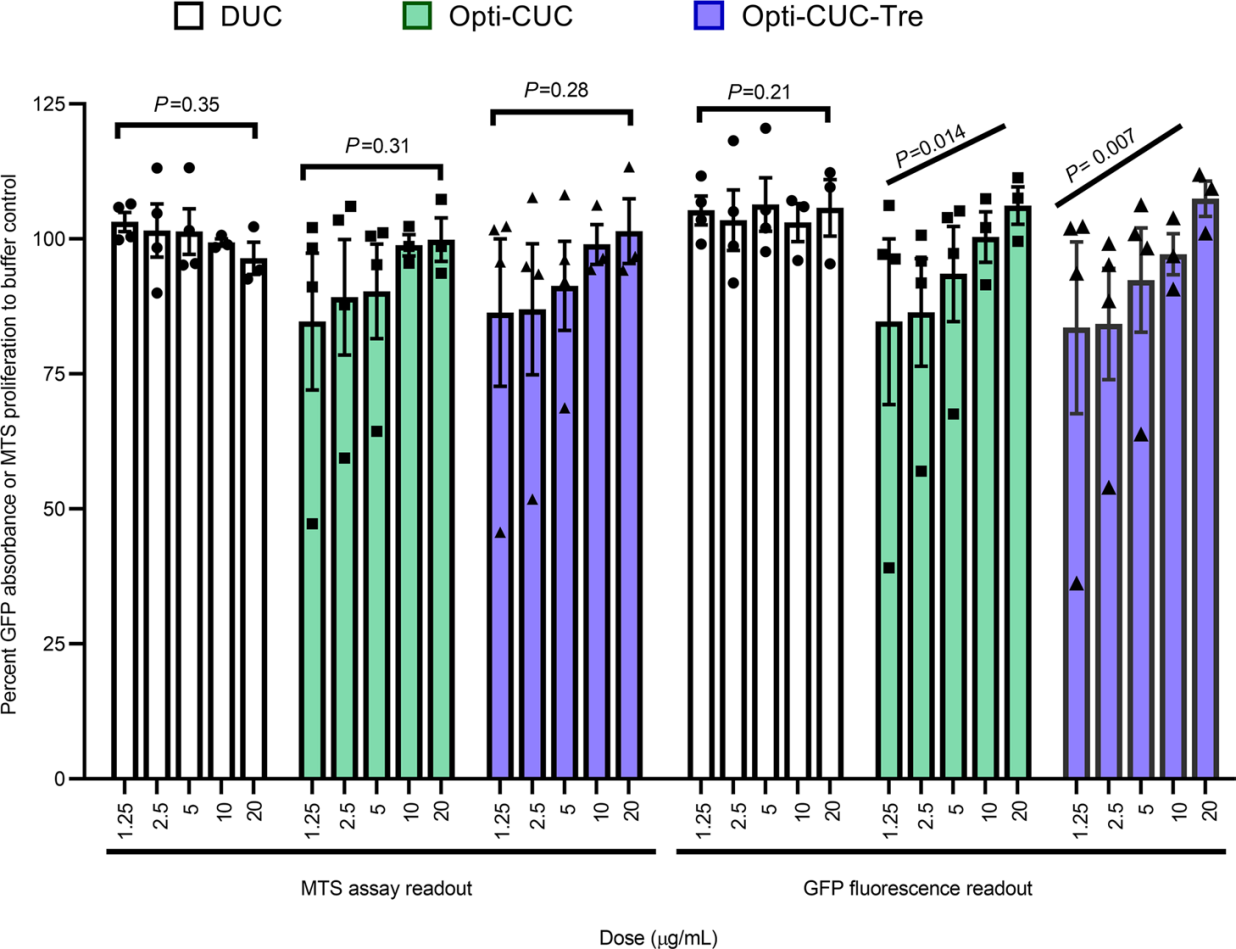
CLL plasma pool–derived EVs promote cell proliferation in vitro. Percentage proliferation change of HS5-GFP stromal cells after 72 hours of incubation with increasing concentrations of EVs using readouts of both the MTS assay and green fluorescence. Data reported as percentage change normalized to control (PBS/Tre buffer). *n* = 4. Data are represented as mean ± SEM. The *P* value (mixed effect model) trend analysis and comparisons are indicated on the graph.

**Figure 9:**
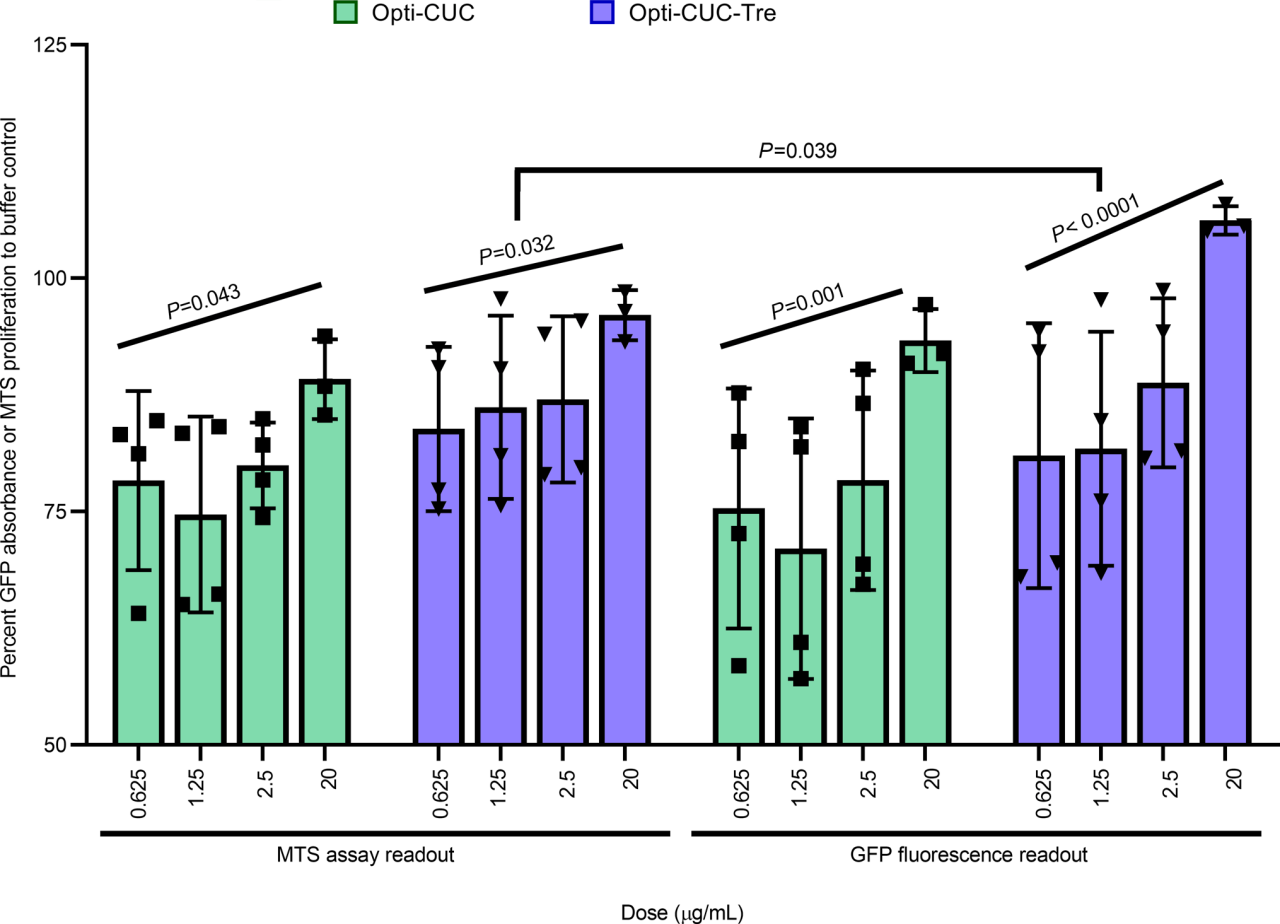
OSU-CLL CLF EVs promote cell proliferation in vitro. Percentage proliferation change of HS-5-GFP stromal cells after 72 hours of incubation with increasing concentrations of EVs using readouts of both the MTS assay and green fluorescence. Data reported as percentage change normalized to control (PBS/Tre buffer). *n* = 4. Data are represented as mean ± SEM. The *P* value (mixed effect model) trend analysis and comparisons are indicated on the graph.

In Figure 8 we show a comparison between GFP and MTS readouts for the assay after 72-hour incubation of the HS5-GFP with increasing doses of EV isolates of CLL-plasma pools (subjected to EV isolation by the 3 methods: DUC, Opti-CUC, and Opti-CUC-Tre) where both readouts showed very comparable patterns (statistical analysis showed no significant differences). With the GFP readout, we saw a dose-dependent increase in cell growth/proliferation for the EVs isolated by Opti-CUC or Opti-CUC-Tre (*P* for trend analysis < 0.05) but not the EVs isolated by DUC. Interestingly, for the MTS readout, although we observed a dose-dependent increase in proliferation, it was not statistically significant (Figure 8), indicating the GFP readout might be more sensitive. In Supplemental Figure 9A we show the GFP absorbance at the 24- and 48-hour time points where we could detect an initial dose-dependent increase in cell growth/proliferation at 24 hours for all 3 isolation methods (*P* for trend analysis < 0.003; Supplemental Figure 9A). At 48 hours, only the Opti-CUC-Tre showed a significant dose-dependent increase (*P* for trend analysis = 0.01; Supplemental Figure 9A). We also performed an MTS assay with regular HS5 cells and found increased proliferation by Opti-CUC and Opti-CUC-Tre versus DUC (Supplemental Figure 9B, *P* < 0.01). Furthermore we performed this assay with OSU-CLL CELLine Flask EV isolates (Figure 9), and the comparison between GFP and MTS readouts also showed no significant differences. A dose-dependent increase in cell growth/proliferation with increased EV does was detected (*P* for trend analysis < 0.05 for MTS readout and *P* < 0.01 for GFP readout, Figure 9).

Our data also emphasize the variability of EV isolates from plasma starting material. This can somewhat be attributed to the level of coenriched lipoproteins (56, 66, 92); since high- and low-density lipoproteins in plasma can aggregate to form larger particles, they become similar in physical characteristics to EVs (68).

## Discussion

Through transfer of bioactive molecules, EVs are recognized as effective signaling mediators (93–95) and offer great potential of becoming off-the-shelf therapies. They can perform therapeutic activities similar to those achieved by their parental cells with the advantages of being non–self-replicating, sterilizing filtration capable, and cryopreservation or lyophilization capable (96, 97).

In this work we aimed to establish a robust and improved workflow for the isolation of EVs from CLL patient plasma and commonly used cell lines MEC1 and OSU-CLL. We raise serious concerns about the use of the serum-free media AIM V as an alternative to EV-depleted, FBS-supplemented media. Here we demonstrated that OptiPrep cushion cannot remove coisolated AIM V media components. This is in accordance with Lamparski et al. (25), who reported that a 30% sucrose/D_2_O (98%) cushion failed to separate these aggregates while ultrafiltration through a 500 kDa NMWCO hollow fiber cartridge could remove them.

Furthermore, we demonstrate that use of bioreactor flasks will minimize the amounts of EV-depleted media required to produce EVs without compromising production or yield. The CELLine AD 1000 (Argos Technologies) flask allows production of EVs supported by half a liter of media while only needing to process 3.5% of that volume, minimizing both spin time and residual media background. This makes the benefit of bioreactors 2-fold: it reduces labor and cost for EV-depleted media preparation and decreases the potential downstream media fingerprint in the EV product.

We show that EV isolation from plasma was improved by combining UC and density gradient cushion to increase purity while utilizing trehalose to enhance yield and reduce aggregation. With the great complexity of the blood proteome, it is essential to utilize techniques to purify blood EVs from soluble proteins and lipoproteins smaller than EVs. Karimi et al. (66) showed by utilizing an iodixanol density cushion followed by SEC column, mass spectrometry identified 800 proteins that had not previously been detected in plasma EVs.

With the diverse nature of EV research, including different starting materials and various downstream applications (biomarker detection, RNA analysis, mass spectrometry, or functional assays), it is unlikely that one method will be able to fit all needs. Quality and integrity of the final EV product do not depend solely on isolation technique but can be affected by other aspects of the workflow. In this work we utilized the natural sugar trehalose, at 25 mM in PBS, to reduce aggregation of EVs and enhance stability (48). We investigated if including trehalose at an earlier stage in the Opti-CUC would enhance purity. We found that it slightly increased P/μg for the EV products.

To our knowledge, this is the first work reporting the use of trehalose in EV isolation from plasma. We demonstrate a slight decrease in protein yield together with a slight increase in P/μg, suggesting a reduction in coisolated contaminants from plasma. We also detect qualitative differences with fewer aggregates and preserved integrity noted on transmission electron microscopy. Addition of trehalose early in the EV isolation workflow for CCM generated from CLF showed a significant increase in P/μg yield for MEC1 EV isolates (*P* = 0.0041) but not for OSU-CLL EV isolates (*P* = 0.433). Although OSU-CLL did not show significant increase in P/μg, it did demonstrate enhanced P/μg in comparison with EV production from conventional T-flasks (*P* = 0.028) while absence of initial trehalose did not (*P* = 0.1132; Figure 7). To this end our data suggest that including trehalose upstream in the EV isolation process can improve the P/μg value of EV isolates and does not show by our evaluation any deleterious effect on surface proteins. Investigating the potential downstream effects of any reagent utilized during the EV isolation protocol has proved essential in light of the recent work by Santucci et al. (42) demonstrating that the use of DTT can promote EV interaction with surrounding macromolecules via disulfide bridges and cause protein aggregations above 37°C.

Furthermore, in this work, we optimize proliferation assays with stroma cells (26, 54) and report the benefit of using HS5-GFP instead of HS-5 to set up these assays. The use of this fluorescent cell line allows monitoring the effect of EVs on the cells at various time points while minimizing the amount of EVs needed. At the terminal time point selected, the MTS reagent can be added to obtain the MTS readout for the proliferation assay. Interestingly GFP readout after 24 hours’ incubation (Supplemental Figure 9) demonstrated an initial increase with all plasma-derived EV products. We speculate this increase might be protein cargo transferred by EVs or non-EV material coisolated from plasma. At 72 hours (Figure 8), we detected for Opti-CUC or Opti-CUC-Tre (but not DUC) a dose-dependent response for the GFP readout that might reflect stromal cell alteration by the transferred bioactive cargo molecules (e.g., mRNAs, miRNAs, and long noncoding RNA). Interestingly, for the Opti-CUC-Tre samples, this dose-dependent response was detected across the 3 time points, while for Opti-CUC, the 48-hour time point was not significant (Supplemental Figure 9).

After in-depth assessment of this workflow by NTA, protein quantification, Western blot, flow cytometry, and electron microscopy, we emphasize that the quality of the final EV product does not merely depend on the selected isolation technique but is greatly affected by other aspects of the workflow, including media selection, culturing vessel selection (standard versus CLF), and downstream buffers/reagents. These results provide reproducible methods to effectively study EVs in CLL and other diseases.

In conclusion, we have assessed in depth a simple workflow capable of generating EVs from CLL patient plasma and cell line with high purity. We show that UC coupled with OptiPrep cushion flotation (Opti-CUC) is a method that provides great purity in an efficient time frame. Together with including trehalose for EV disaggregation and cryoprotection, we have increased yield and integrity of EV products. Even with more sophisticated and laborious EV purification methods, the coenrichment of lipoproteins cannot be overcome (66), so we also demonstrate an optimized proliferation-based, functional assay to serve as a robust quality control assessment tool before proceeding with downstream applications.

## Methods

In this work we have adhered to the MISEV guidelines (62, 98) when possible. We have submitted all relevant data of our experiments to the EV-TRACK (99) knowledge base (EV-TRACK ID: EV200004).

### Patient sample processing

Peripheral blood from CLL patients recruited at the OSU Comprehensive Cancer Center was collected into BD Vacutainer tubes containing Acid Citrate Dextrose (Solution A, 364606). All patients examined had CLL as defined by the 2008 International Workshop on Chronic Lymphocytic Leukemia criteria (100). After collection, whole blood was centrifuged for 10 minutes at 300*g* (room temperature) to separate plasma. The plasma was subjected to 2 more spins at 2500*g* for 15 minutes (room temperature) to obtain platelet-free plasma (55, 56). Plasma was stored at −80°C until further use.

### EV isolation from plasma

EVs were isolated from thawed plasma after diluting with at least an equal volume of PBS (9). DUC was performed as previously described (8, 9). Briefly, thawed plasma was spun at 2000*g* for 20 minutes at 4°C to remove any debris generated during thawing. Plasma was then subjected to centrifugation at 10,000*g* for 30 minutes (4°C), followed by 100,000*g* for 70 minutes (4°C) in a fixed-angle rotor (Beckman Type 70 Ti) in 26.3 mL polycarbonate tubes (Beckman Coulter). All washes and final pellet recovery were done with 25 mM trehalose (BP268725, Fisher BioReagents) in PBS (14190144, Gibco; ref. 48). This is referred to throughout this work as PBS/Tre buffer. For the samples being processed by the Opti-CUC method, the samples were processed similar to DUC with the following modifications (26, 27): after the first 100,000*g* spin the pellet was resuspended in 4 mL PBS/Tre. This was underlaid with 1 mL of an OptiPrep cushion (Axis-Shield, 17%) in a 5 mL, thin-wall, ultraclear tube (Beckman Coulter). The samples were ultracentrifuged at 100,000*g* at 4°C for 75 minutes in a swinging bucket rotor (Beckman Coulter type 55 Ti). The cushion and interphase were collected and washed with PBS/Tre in a final 100,000*g* spin for 70 minutes (4°C) in a fixed-angle rotor (Beckman Coulter type 70 Ti) in 26.3 mL polycarbonate tubes (Beckman Coulter). The EV pellet (Opti-CUC isolate) was recovered in PBS/Tre. Volumes of starting material and final EV product were recorded for calculations. For some samples, the supernatant above the cushion (Figure 1A) was processed (pelleted supernatant) for further analysis. For the plasma pool, individual samples (previously at −80°C) were thawed, then pooled to achieve a volume of approximately 100 mL/pool. This pooled plasma was divided between the 3 workflows. For the DUC portion, the plasma was diluted with an equal volume of PBS and processed by standard DUC procedure. For the Opti-CUC portion, plasma was also diluted with an equal volume of PBS and allowed to rock on ice for 20 minutes before being processed with the Opti-CUC method as described above. The third portion, for Opti-CUC-Tre, was processed exactly like the Opti-CUC but instead of initial dilution 1:1 with PBS, it was diluted with an equal volume of 50 mM trehalose in PBS, thus having a final trehalose concentration of 25 mM.

### Culture media

Cells were cultured in RPMI 1640 medium (purchased from Gibco), supplemented with l-glutamine (2 mM), penicillin (56 IU/mL), and streptomycin (56 μg/mL). DMEM was also purchased from Gibco and similarly supplemented with l-glutamine, penicillin, and streptomycin. Then 10% FBS was added for MEC1 (70) and 20% FBS for OSU-CLL (71). For EV production, cells were washed in PBS and switched to EV-depleted complete RPMI or the serum-free media AIM V (purchased from Gibco). For depletion of media (RPMI or DMEM), the complete medium was subjected to UC at 110,000*g* for 18 hours (4°C; refs. 27, 101, 102) in a fixed-angle rotor (Beckman Coulter 70 Ti) in 26.3 mL polycarbonate tubes (Beckman Coulter).

### Cell culture and EV isolation from standard culture T-flasks

The CLL cell lines OSU-CLL and MEC1 were utilized in this study with routine testing for mycoplasma contamination. MEC1 (70) was obtained from DSMZ (DACC 497); OSU-CLL was generated in our lab (71). For EV production, cells were seeded in standard culture T-flasks at 0.8 million/mL in EV-depleted complete RPMI or AIM V. After 72 hours conditioned culture media were harvested, then centrifuged for 10 minutes at 300*g* (4°C) to remove cells. Supernatants were collected and EVs were isolated by DUC (for RPMI cultures only) or Opti-CUC (for RPMI and AIM V cultures) as described above. To prepare unconditioned media samples, 100 mL of unconditioned media were processed in the same manner described above. A total of 6 replicates per cell line or unconditioned media were prepared.

### Cell culture and EV isolation from CELLine reactors

CELLine AD 1000 flasks (Argos Technologies) were utilized in this work. A total of 25 × 10^6^ cells suspended in 17 mL of EV-depleted complete RPMI media were seeded in the inner compartment of the flasks. A total of 500 mL of complete media was added to the outer compartment (14, 27, 102). These compartments are separated by a 10 kDa semipermeable membrane, which allows continuous diffusion of nutrients and removal of wastes (27). Weekly the culture medium in the inner compartment was collected for processing (1 replicate), with outer compartment media replaced with 500 mL fresh complete RPMI and inner compartment reseeded with 7 × 10^6^ cells in 17 mL EV-depleted complete RPMI media. The weekly cell suspension harvest of the inner compartment was processed as described (26, 27) to isolate EVs. Each harvest was divided into two, one processed by Opti-CUC and other by Opti-CUC-Tre. For Opti-CUC-Tre samples, before the 2000*g* step, trehalose was added to a final concentration of 25 mM (using a 250 mM solution of trehalose in PBS). For the Opti-CUC sample, an equal volume of PBS was added. For both samples, after the addition of the 250 mM trehalose (for Opti-CUC-Tre) or PBS (for the Opti-CUC), the samples were rocked on ice for 20 minutes before proceeding with the EV isolation steps. All washes and final pellet recovery were done with PBS/Tre. A total of 6 replicates per cell line were prepared.

### Characterization of EVs

#### Protein quantification.

Protein quantification of EVs was conducted without use of detergent using the Qubit Protein Assay Kit (Invitrogen) (69) as recommended by the manufacturer, and concentrations were read using a Qubit 3.0 fluorometer (56, 69, 103).

#### Nanoparticle tracking analysis.

Size and concentration of isolated exosomes were measured by NanoSight NS300 (Malvern Instruments) at the OSU Comprehensive Cancer Center (OSUCCC) Analytical Cytometry Shared Resource as previously described (8). Briefly samples were diluted to obtain a concentration within the range of 10^8^ to 10^9^ P/mL. Analysis was carried out with the NTA software (version 3.3 build 3.3.104) with a syringe pump speed of 25. Camera level was set to 12, and detection threshold was set to 5. All NTA frequency size distribution graphs plotted in the manuscript were for an EV product concentration of 500×.

#### Western blot.

Western blots were performed as described (69) with some modifications. Briefly, EVs were lysed in RIPA buffer with 1 mM PMSF; after 30 seconds’ brief sonication, samples were heated at 95°C for 5 minutes in sample loading buffer: reducing (0.1 M Tris-HCl at pH 6.8, 5% glycerol, 1.5% SDS, 1.3% DTT, and 0.04% bromophenol blue) or nonreducing sample buffer (without DTT). EVs were resolved by SDS-polyacrylamide gel electrophoresis, then transferred to nitrocellulose membranes and blotted with specific primary and secondary antibodies. Antibodies used for immunoblotting were purchased from Santa Cruz Biotechnology: CD63 (MX-49.129.5, sc-5275, nonreducing conditions), Calnexin (AF18, SC-23954), CD9 (ALB 6, sc-59140), HSP70 (W27, sc-24), Alix (1A12, sc-53540), TSG 101 (51, sc-136111), Albumin (F-10, sc-271605), CD81 (B-11, sc-166029), GAPDH (0411, sc-47724). All primary antibodies were diluted 1:1000, except GAPDH was diluted 1:30,000 and secondary antibody (m-IgGκ BP-HRP, sc-516102, diluted 1:2000). Protein bands were detected using Pierce ECL Western Blotting Substrate (Thermo Fisher Scientific) on x-ray film.

#### Bead-based flow cytometry.

We performed bead-based flow cytometry as described (69). Briefly, a volume of EV containing 10 μg protein was incubated with 10 μL latex beads at room temperature for 15 minutes with continuous rotation. Then 1 mL Dulbecco’s PBS was added and they were left to rotate overnight at 4°C. Beads were then incubated for 30 minutes with glycine (100 mM final). The samples were washed with PBS/0.5% BSA. Staining was performed at 4°C for 30 minutes using fluorophore-coupled primary antibodies or isotype controls and analyzed. Samples were analyzed on an LSRFortessa flow cytometer (BD). Voltage was adjusted to detect beads from the dot plot of forward and side scatter, and only single beads were gated for fluorescence signal analysis. Data obtained from the specific antibody and isotype control of each sample were analyzed and compared with Kaluza software (Beckman Coulter). Delta mean fluorescence of samples was calculated by subtracting MFI of each sample from its isotype control. These antibodies or isotype controls were purchased from BD Pharmingen, BioLegend, or eBioscience (Thermo Fisher Scientific) as follows: CD235A (mouse IgG2b kappa, FITC, antibody: 11-9987-82, isotype: 11-4732-82, eBioscience); CD9 (mouse BALB/c IgG1, kappa, PE, antibody: 555372, isotype: 555749, BD); CD45 (mouse IgG1, kappa, APC, antibody: 555485, isotype: 555751, BD); CD19 (mouse IgG1, kappa, PE, antibody: 555413, isotype: 555749, BD); CD81 (mouse IgG1, kappa, PerCP/Cy5.5, antibody: 349507, isotype: 400149, BioLegend); CD52 (mouse IgG2b, kappa,PE/Cy7, antibody: 316011, isotype:400325, BioLegend); HLA-DR (mouse IgG2a, kappa, Pacific Blue, antibody: 307624, isotype: 400235, BioLegend). All antibodies were utilized at the dilution suggested by the manufacturer.

#### Transmission electron microscopy.

Purified EVs were observed by transmission electron microscopy. A 15 μL sample drop was placed on a formvar/carbon 200 mesh copper grid (01800, Ted Pella) for 5 minutes. The sample was then removed by blotting the grid with filter paper and subsequently washed 3 times with distilled water. Grids were then stained with 2% uranyl acetate (22400-2, Electron Microscopy Sciences) for 5 minutes, blotted dry with filter paper, and further left to air-dry for 10 minutes. The samples were observed under a JEOL JEM 1010 transmission electron microscope and images collected using a MegaView III camera and iTEM imaging software.

#### Proliferation assay.

HS-5 (104) was obtained from ATCC (CRL-11882), and HS5-GFP was a gift from William Dalton’s Laboratory (H. Lee Moffitt Cancer Center, Tampa, Florida, USA). Both cell lines were authenticated and maintained in 10% RPMI with routine testing for mycoplasma contamination. The proliferation assay was developed from the assay reported (26) with several modifications. HS5-GFP or HS-5 cells were maintained in 10% RPMI. To set up the assay, cells were washed in serum-free DMEM, then suspended in 2% DMEM (EV depleted). Cells were seeded into clear, flat-bottom, 96-well plates. A total of 5500 cells were delivered in 80 μL of 2% DMEM (EV depleted) to each well. A total of 20 μL of either EV dose or buffer control (PBS/Tre) was added to each well such that final volume was 100 μL. No cells were seeded in the peripheral wells (but instead PBS) to avoid inconsistency due to liquid evaporation from the outer wells (105). Plates were incubated in a hypoxia incubator (1.5% O_2_, 5% CO_2_; Thermo Fisher Scientific, Heracell VIOS 160i CO_2_ incubator). For assays set up with HS5-GFP, the GFP absorbance was recorded every 24 hours with a microplate reader (DTX 880) at Ex/Em of 485/530 nm. When indicated, CellTiter 96 Aqueous MTS reagent (Promega) was added to cells followed by a 3-hour incubation in a normoxia incubator (5% CO_2_, 20 % O_2_); then absorbance was measured at 490 nm using a DTX 880 plate reader to obtain the MTS readout. For assays set up with regular HS-5, after 96-hour incubation in the hypoxia incubator, the MTS reagent was added. All MTS assay data and GFP absorbance were represented as percentage proliferation normalized to buffer control.

### Statistics

For experiments involving 2 independent groups, the difference between them was tested by 2-sample 2-tailed *t* tests. For the experiments to compare different conditions by using the same sample/pool, paired 2-tailed *t* test (2 conditions) or 2-way ANOVA with repeated measures (>2 conditions) was conducted. Normality assumption was tested before data analysis; log transformation was applied when data were not normally distributed. Multiplicity was adjusted by Holm’s procedure, and data analysis was conducted in SAS 9.4 (SAS, Inc). In this work, we considered *P* values 0.05 and below as significant.

### Study approval

Blood was drawn from patients after receipt of written informed consent under a protocol approved by the Institutional Review Board of The Ohio State University in accordance with the Declaration of Helsinki.

## Author contributions

JCB, KTL, and SE conceived and designed the study. SE developed methodology. SE, EJS, JCB, KTL, SAB, JAW, and KAR were responsible for acquisition of data (provided animals, acquired patients, managed patient care, provided facilities, etc.). SE, EJS, EC, XMM, ARB, and EPC were responsible for analysis and interpretation of data (e.g., statistical analysis, biostatistics, computational analysis). SE, EC, AJJ, JCB, KTL, KAR, and EJS were responsible for writing, review, and/or revision of the manuscript. AJJ, KTL, and JCB supervised the study. NM provided scientific input.

## Supplementary Material

Supplemental data

## Version 1 08/09/2021

Electronic publication
