# Supplementary material for: Optimizing extracellular vesicles’ isolation from chronic lymphocytic leukemia patient plasma and cell line supernatant
Source: JCI Insight. 2021 Aug 9;6(15):e137937. doi: 10.1172/jci.insight.137937 (PMC8410027; doi:10.1172/jci.insight.137937)
Supplement: Supplemental data [file jciinsight-6-137937-s242.pdf]

Supplementary Figures

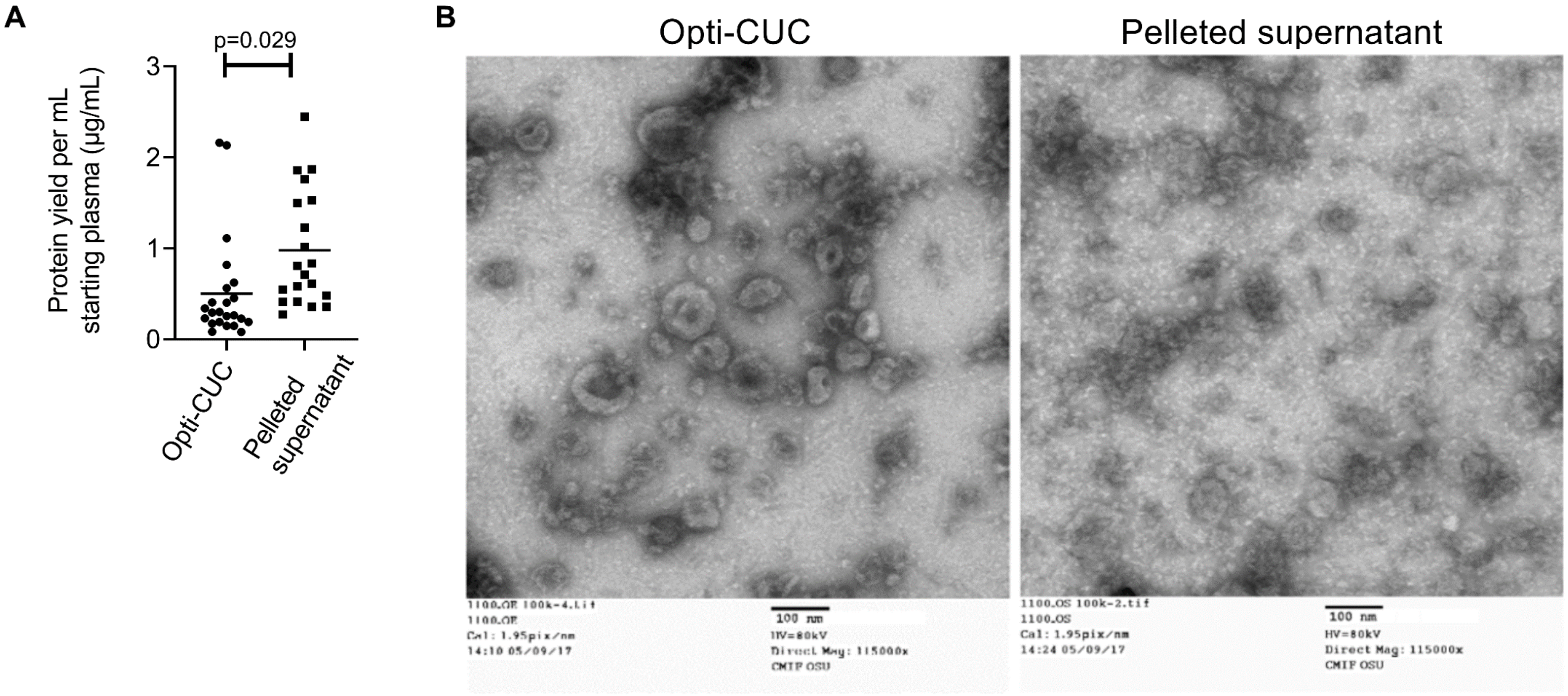

Figure S1. CLL-plasma derived EVs by Opti-CUC (A) Protein yield per mL starting plasma volume (µg/mL) detected in Opti-CUC EV isolate and the pelleted supernatant for Opti-CUC EV isolates shown in Figure. 1B (n=22, p=0.029, paired t-test), horizontal line represents mean (B) Representative electron microscopy images comparing Opti-CUC EV isolate and the pelleted supernatant. Scale bar 100 nm.

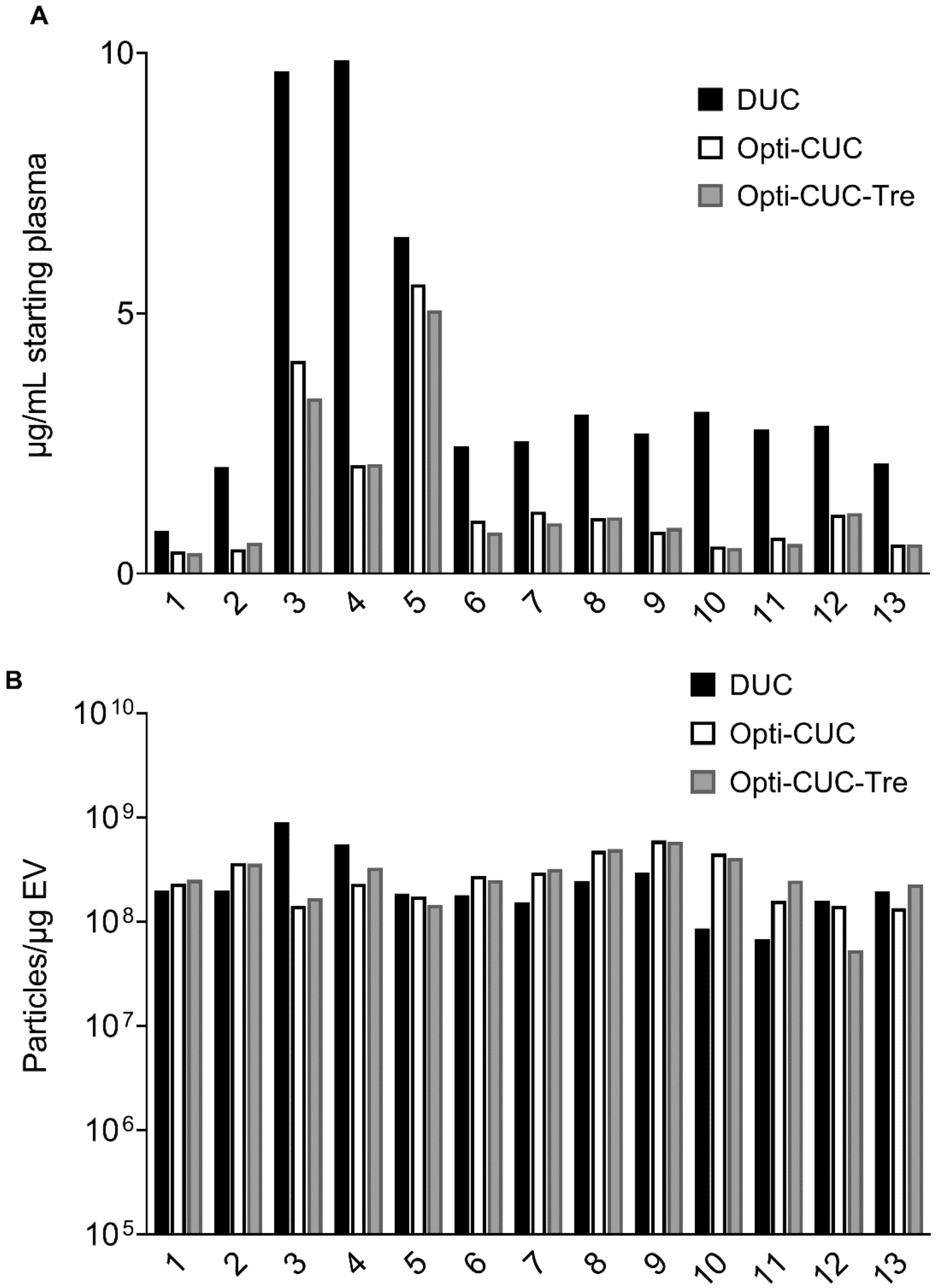

Figure S2. Protein yield (μg) per mL starting plasma volume (A) and Particle/μg value (B) for the 13 plasma pool samples that are averaged in Figure 2B. The plasma pools were isolated by DUC, Opti-CUC or Opti-CUC-Tre.

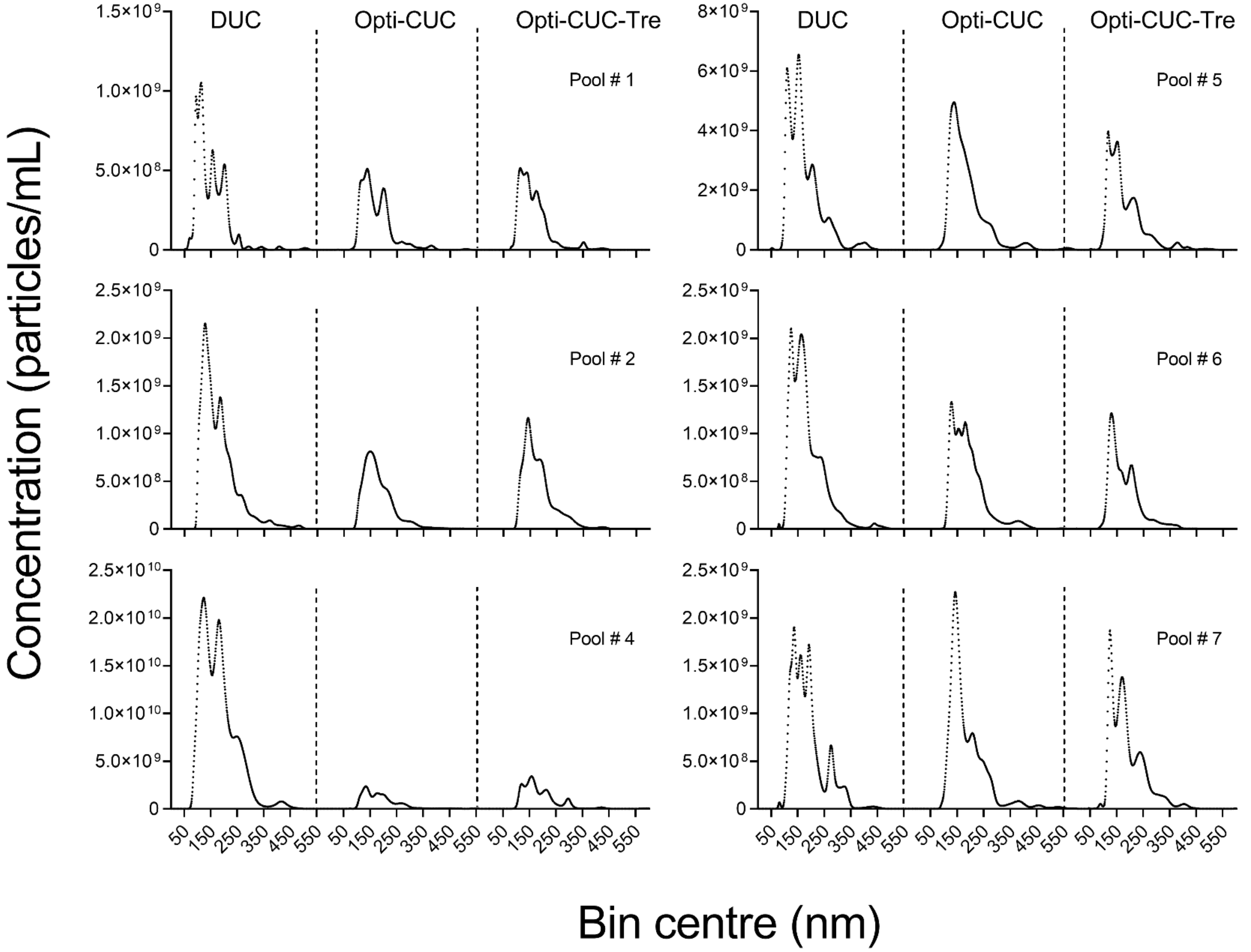

Figure S3. Isolated particles subjected to nanoparticle tracking analysis (NTA) measurement for concentration and size distribution, a representative plot presented is the average of three 30-second videos for 6 of the plasma pool sets discussed in Figure 2. The plasma pools were isolated by DUC, Opti-CUC or Opti-CUC-Tre.

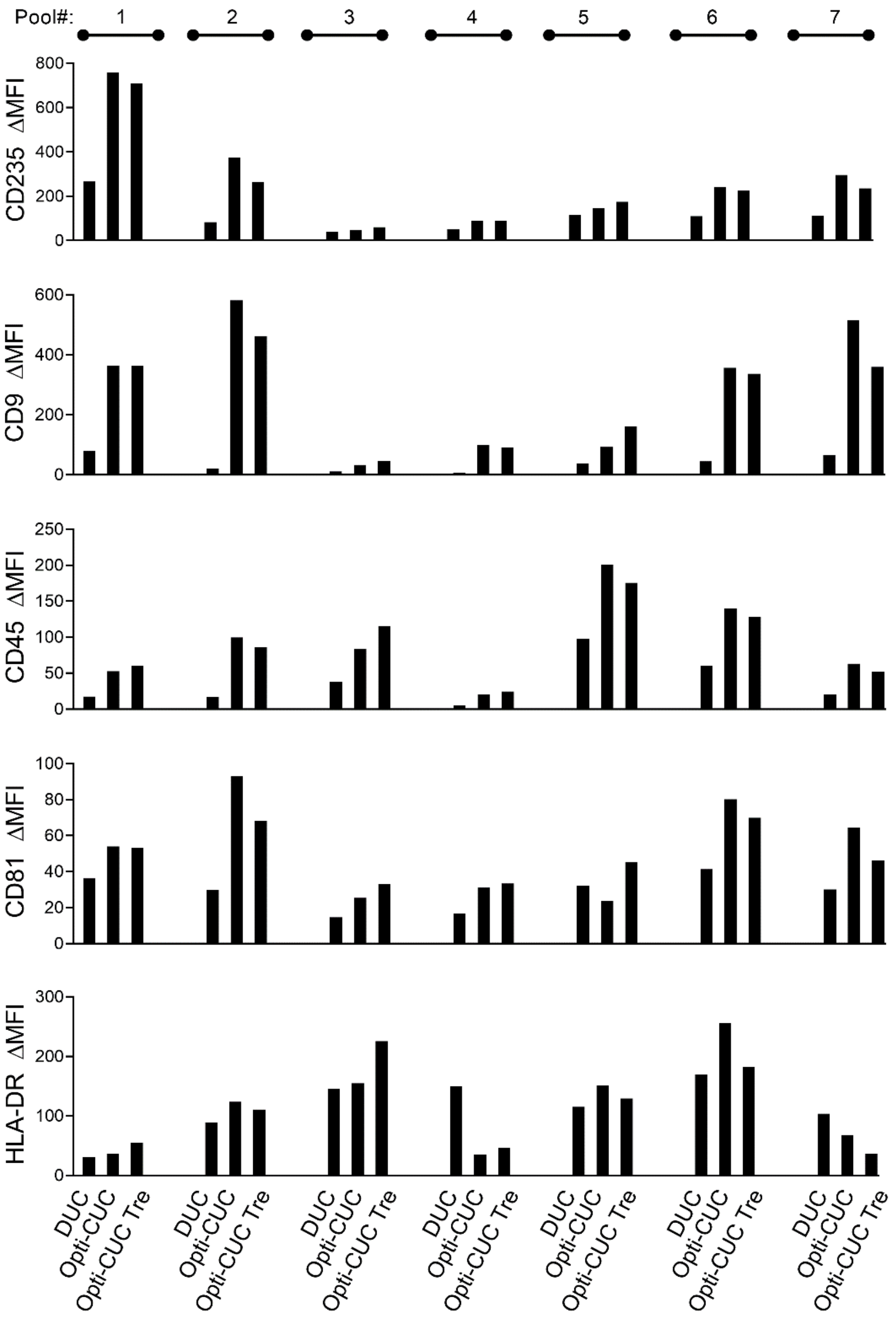

Figure S4. Bead-based flow cytometry analysis of EV isolates from the individual plasma pool sets that are averaged in Figure 2D . Delta median fluorescence of samples calculated by subtracting median fluorescence intensity (MFI) of each sample from its isotype control.

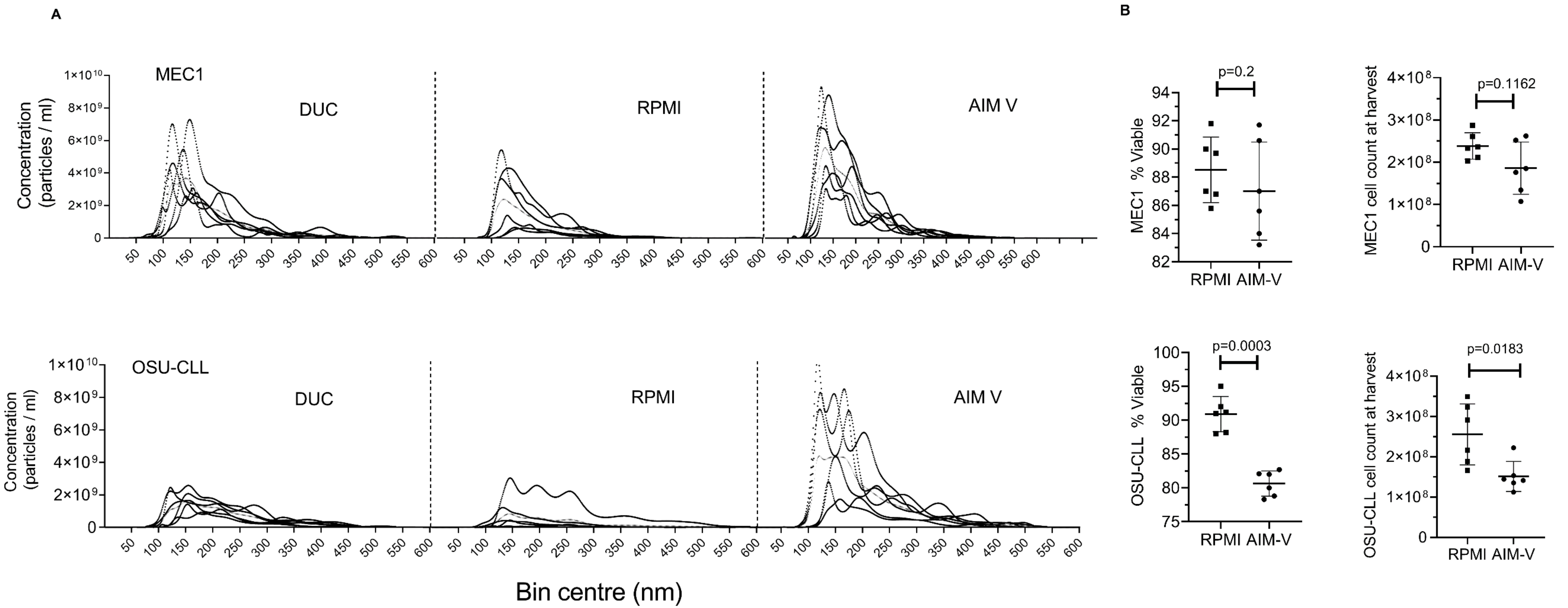

Figure S5. EV isolation from MEC1 and OSU-CLL in standard flasks cultured in AIM V or RPMI (A) Isolated particles subjected to nanoparticle tracking analysis (NTA) measurement for concentration and size distribution, a representative plot presented is the average of three 30-second videos for MEC1 (upper panel) and OSU-CLL (lower panel) for the different media, n=6. Further data for these samples in Figure 3. (B) Viability and total cell count at harvest for MEC1 or OSU-CLL cultured in 100 mL RPMI or AIM V, n=6, paired t-test, data are represented as mean ± SD. RPMI= EV depleted complete RPMI.

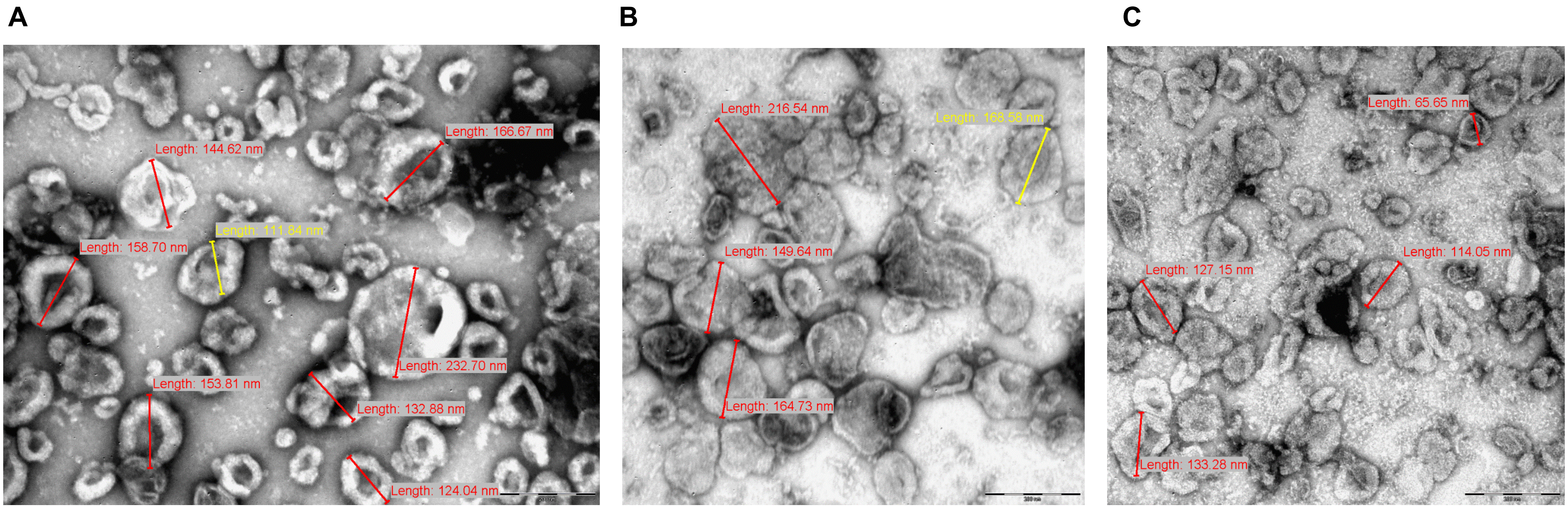

Figure S6. Representative Transmission Electron Microscopy (TEM) images with size annotations of MEC1 EVs, scale bar 200 nm. (A) MEC1 cultured in standard flask in RPMI isolated by Opti-CUC. (B and C) CeLLine Flask EVs isolated by Opti-CUC in absence (B) or presence (C) of initial trehalose. RPMI= EV depleted complete RPMI.

A

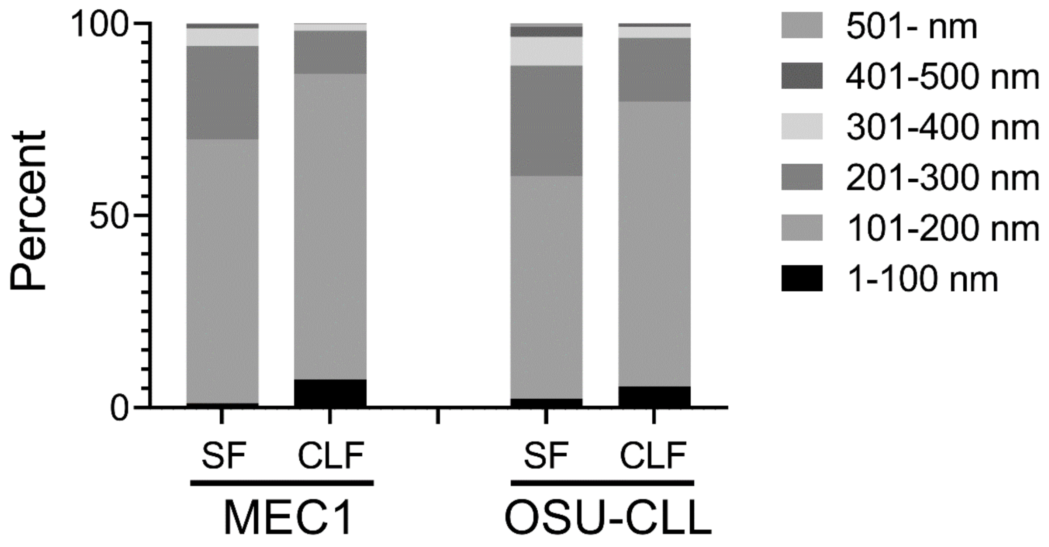

B

Bin nm range		1-100	101-200	201-300	301-400	401-500	501 -
MEC1	SF-EV	1.13	68.65	24.38	4.67	0.98	0.2
	CLF-EV	7.31	79.62	11.16	1.68	0.18	0.04
	t-test	**<.0001**	**0.0194**	**0.0014**	**0.0208**	**0.0211**	0.0837
OSU-CLL	SF-EV	2.32	58.06	28.62	7.52	2.67	0.81
	CLF-EV	5.5	74.2	16.56	2.92	0.75	0.07
	t-test	0.0516	**0.0028**	**0.0019**	**0.0089**	0.1093	**0.0466**

$p \leqslant 0.05$ in bold

Figure S7. Size comparisons of extracellular vesicles (EVs) measured with nanoparticle tracking analysis (NTA) and analyzed by bins of size classes, (n=6) for cells cultured in standard flasks (SF) versus CLF (CeLLine Flask) no trehalose condition. For both flasks, cells were cultured in EV-depleted RPMI and EVs were isolated by Opti-CUC. (A) Segmented Bar Graph showing mean percentage per size bin (B) 2 tailed t-test statistical comparisons between the SF-EVs and the CLF-EVs for each bin, $p \leq 0.05$ in bold.

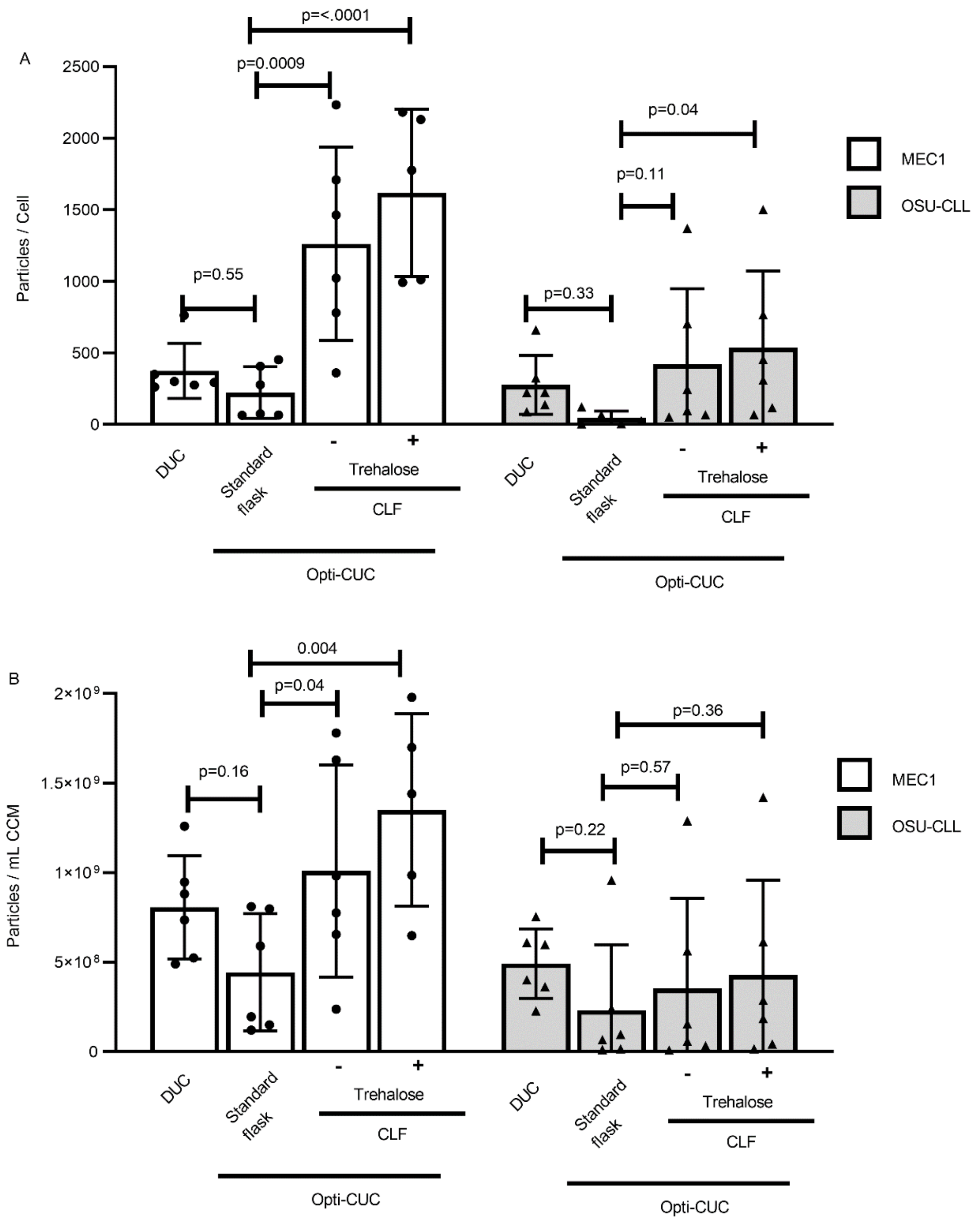

Figure S8. A summary plot showing P/cell values (A) and P/mL CCM (B) for all samples reported in this work that were cultured in RPMI (n=6, ANOVA with repeated measures). Data are represented as mean ± SD. RPMI= EV depleted complete RPMI, CLF= CeLLine Flask, CCM=Conditioned Culture Media.

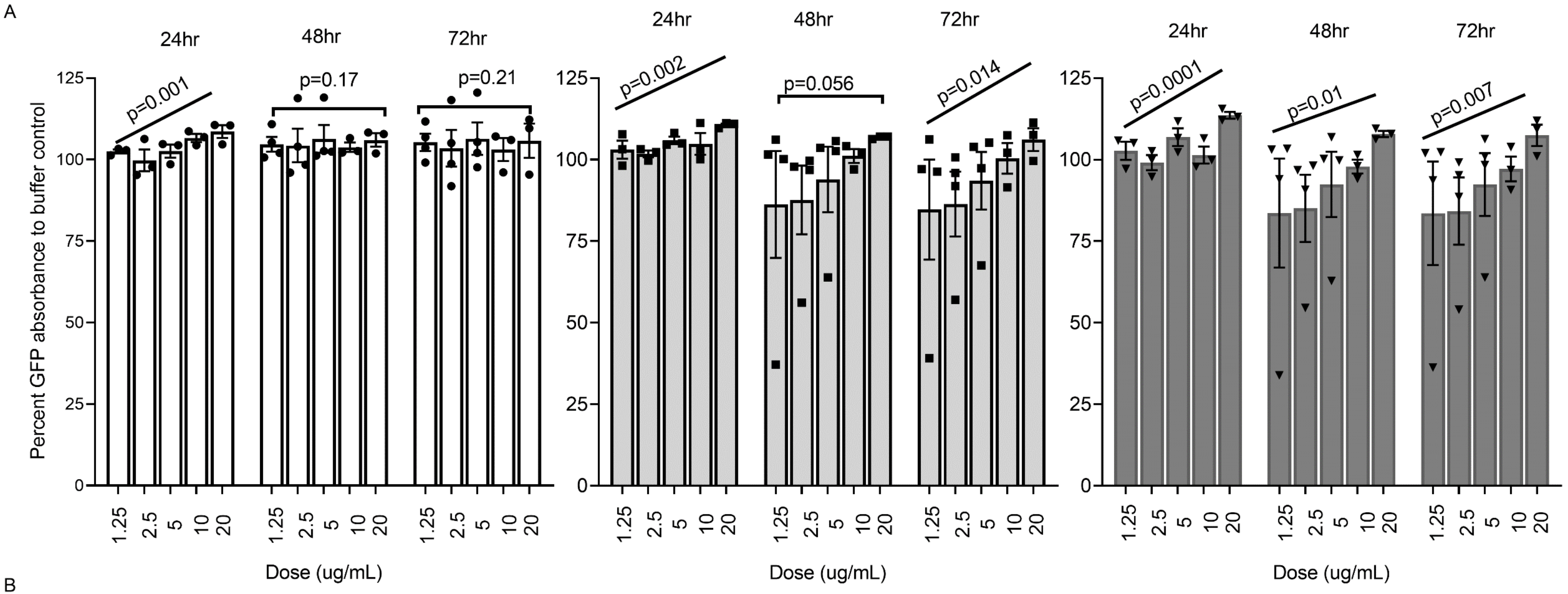

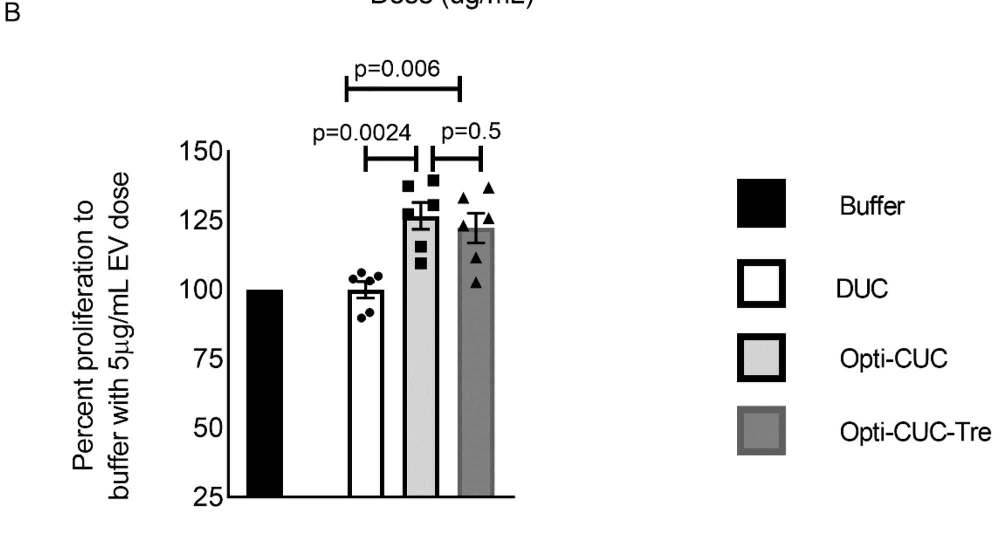

Figure S9. CLL-plasma pool- derived EVs promote cell proliferation in vitro. A) Comparison of percent proliferation change of HS-5-GFP stromal cells based on green fluorescence readout after incubation with increasing concentrations of EVs derived from CLL-plasma pools at 24hr, 48hr and 72hr. The 72hr data is repeated from figure 8 for comparison. Data reported as percent change normalized to control (PBS/Tre buffer), n=4, data are represented as mean ± SEM. The p-value (mixed effect model) trend analysis are indicated on the graph. B) Percent proliferation change of HS-5 stromal cells after 96 hours of incubation with increasing concentrations of CLL-plasma pool-derived EVs using MTS assay. Data reported as percent proliferation change normalized to control (PBS/Tre buffer), n=6, data are represented as mean ± SEM.

Full unedited gel for Figure 2-C

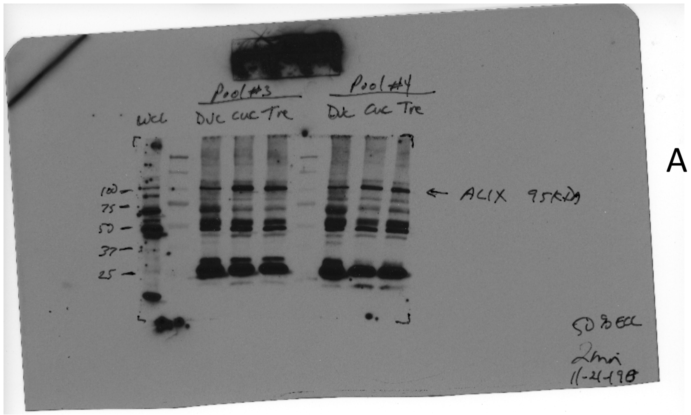

ALIX

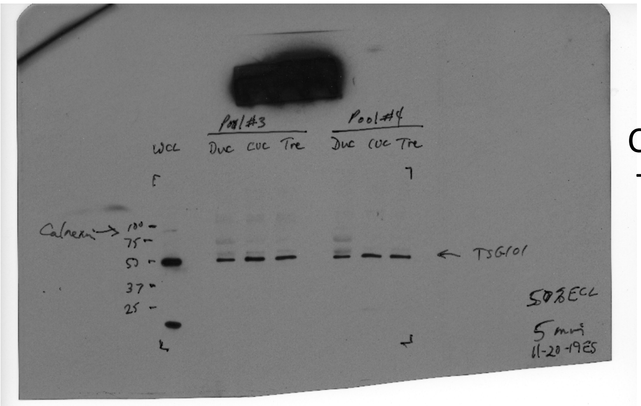

Calnexin
TSG101

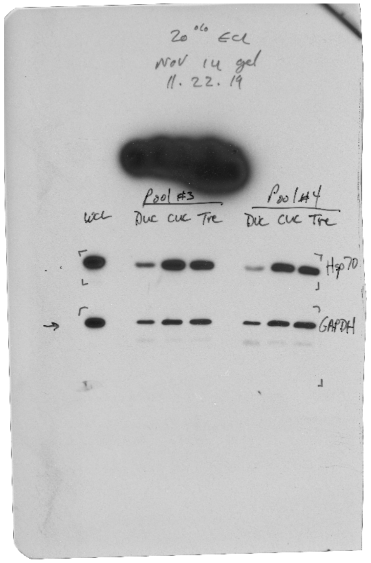

GAPDH

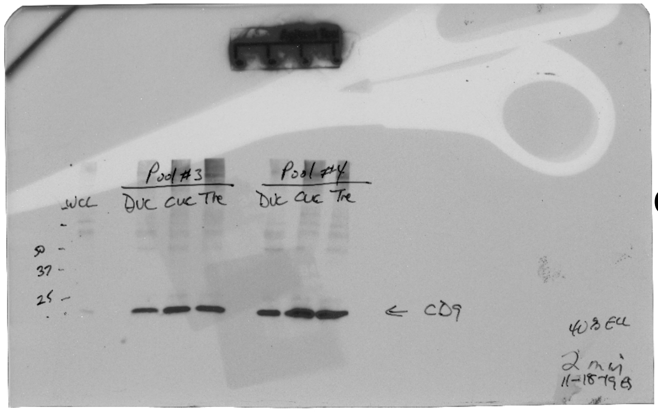

CD9

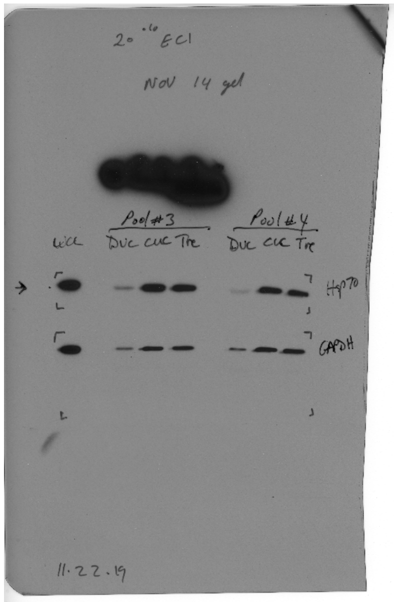

HSP70

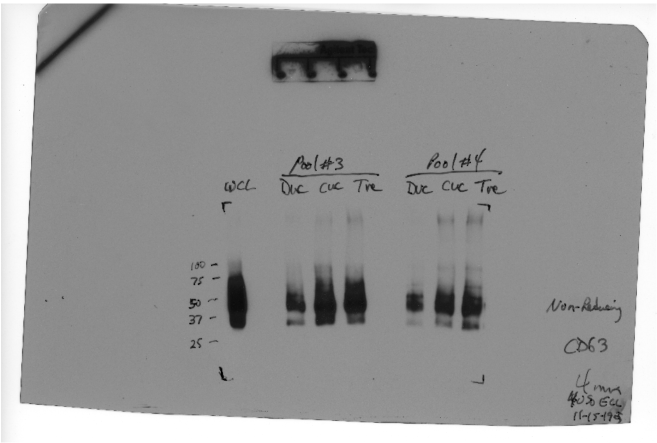

CD63

Full unedited gel for Figure 6-A, Set 1&2

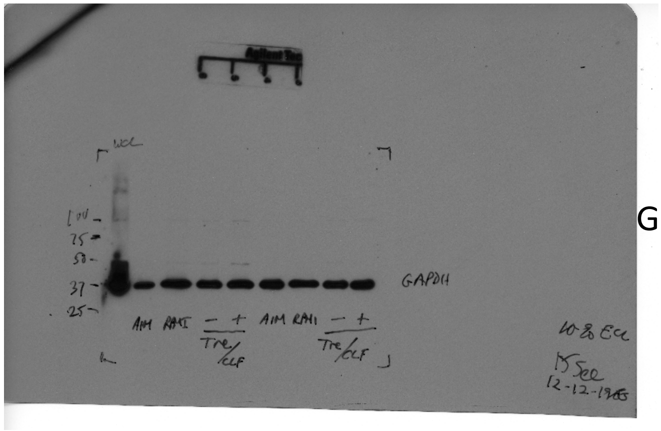

GAPDH

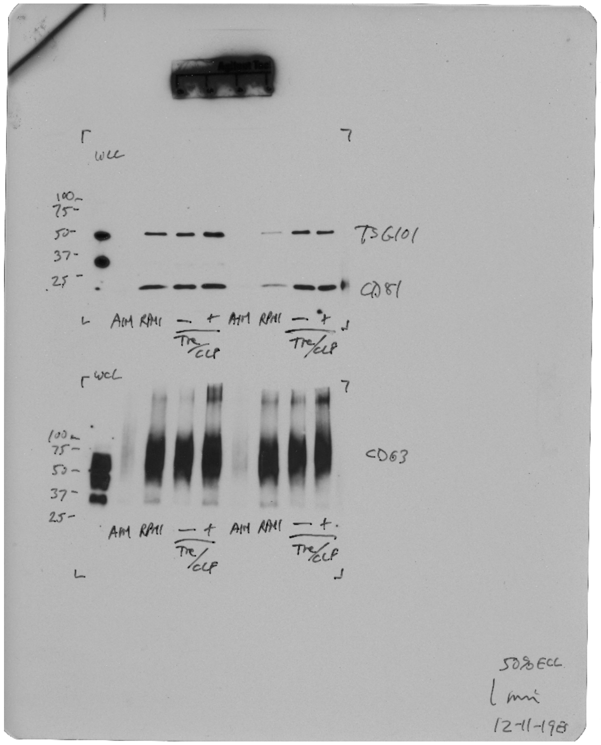

TSG101
CD81
CD63

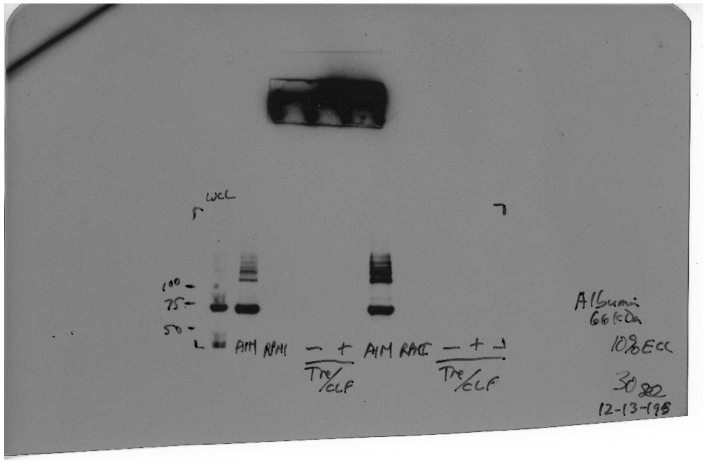

Albumin

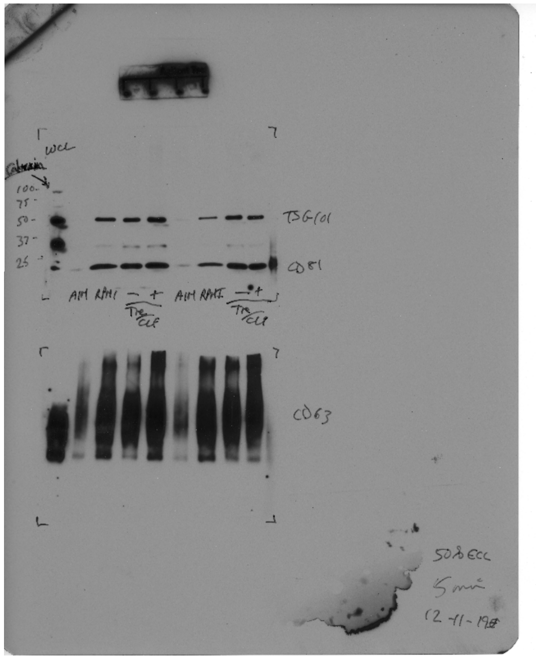

Calnexin

Full unedited gel for Figure 6-A, Set 3&4

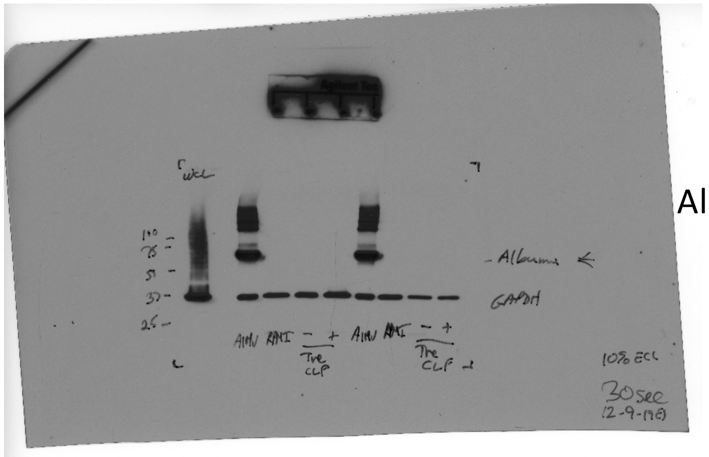

Albumin

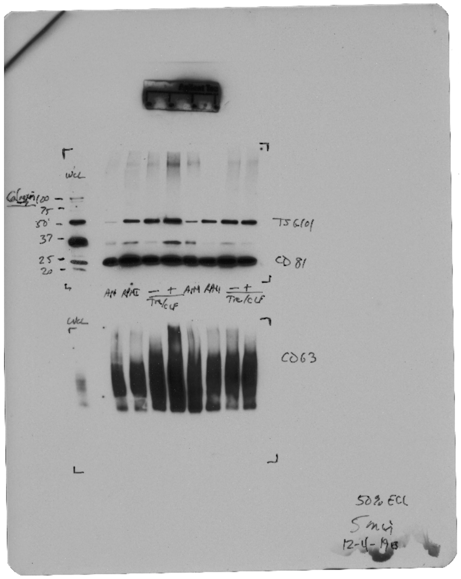

Calnexin

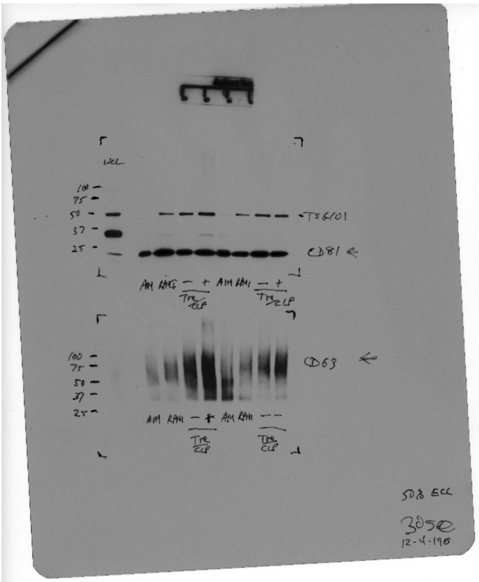

CD81

CD63

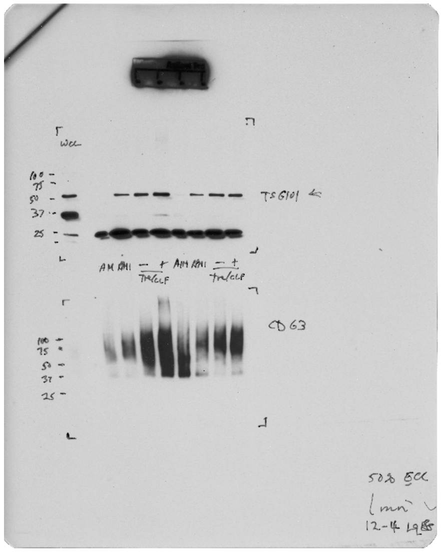

TSG101

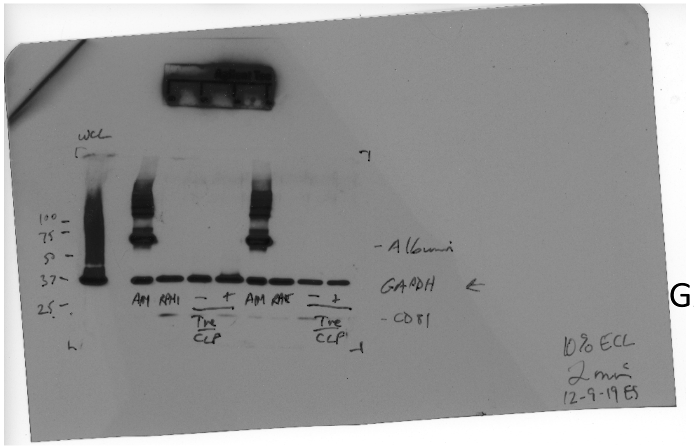

GAPDH
